# DHP-Derivative and Low Oxygen Tension Effectively Induces Human Adipose Stromal Cell Reprogramming

**DOI:** 10.1371/journal.pone.0009026

**Published:** 2010-02-09

**Authors:** Min Ki Jee, Ji Hoon Kim, Yong Man Han, Sung Jun Jung, Kyung Sun Kang, Dong Wook Kim, Soo Kyung Kang

**Affiliations:** 1 Department of Biotechnology, College of Veterinary Medicine, Seoul National University, Seoul, Korea; 2 Department of Biological Sciences, Korea Advanced Institute of Science and Technology (KAIST), Daejeon, Korea; 3 Department of Veterinary Public Health, Laboratory of Stem Cell and Tumor Biology, College of Veterinary Medicine, Seoul National University, Seoul, Korea; 4 Department of Physiology, College of Medicine, Yonsei University, Seoul, Korea; 5 Department of Physiology, College of Medicine, Han Yang University, Seoul, Korea; The University of Hong Kong, China

## Abstract

**Background and Methods:**

In this study, we utilized a combination of low oxygen tension and a novel anti-oxidant, 4-(3,4-dihydroxy-phenyl)-derivative (DHP-d) to directly induce adipose tissue stromal cells (ATSC) to de-differentiate into more primitive stem cells. De-differentiated ATSCs was overexpress stemness genes, Rex-1, Oct-4, Sox-2, and Nanog. Additionally, demethylation of the regulatory regions of Rex-1, stemnesses, and HIF1α and scavenging of reactive oxygen species were finally resulted in an improved stem cell behavior of de-differentiate ATSC (de-ATSC). Proliferation activity of ATSCs after dedifferentiation was induced by REX1, Oct4, and JAK/STAT3 directly or indirectly. De-ATSCs showed increased migration activity that mediated by P38/JUNK and ERK phosphorylation. Moreover, regenerative efficacy of de-ATSC engrafted spinal cord-injured rats and chemical-induced diabetes animals were significantly restored their functions.

**Conclusions/Significance:**

Our stem cell remodeling system may provide a good model which would provide insight into the molecular mechanisms underlying ATSC proliferation and transdifferentiation. Also, these multipotent stem cells can be harvested may provide us with a valuable reservoir of primitive and autologous stem cells for use in a broad spectrum of regenerative cell-based disease therapy.

## Introduction

Although the classic definition of cell plasticity from stem cell biology specifies the ability of stem cells to differentiate into a variety of cell lineages, the term is also currently applied to the ability of a given cell type to reciprocally dedifferentiate, re-differentiate, and/or trans-differentiate in response to specific stimuli [1], [2]. Cellular de-differentiation underlies contemporary topical issues in stem cell biology, most notably regeneration and nuclear cloning. In stem cell biology, this process characterizes the transition of differentiated somatic cells to pluripotent stem cells, and is accompanied by global chromatin reorganization, which is itself associated with the reprogramming of gene expression. De-differentiation signifies the withdrawal of cells from a given differentiated state into a stem cell-like state, which confers pluripotency, a process that precedes re-entry into the cell cycle [3]. The state of de-differentiation can be determined by changes in cell morphology, genome organization, and the gene expression pattern, as well as by the capability of protoplasts to differentiate into multiple types of cells, depending on the type of applied stimulus [4]–[7]. Histone methylation activity is required for the establishment and maintenance of the de-differentiated state and/or re-entry into the cell cycle. The complexity of cellular de-differentiation, and particularly the occurrence of DNA recombination, can result in genome instability [8]. Several studies have demonstrated that freezing-induced and traumatic CNS-induced injuries facilitate the appearance of some radial glia-like fibers, which express Nestin in adult rodents [9]–[14]. A variety of transitional forms of cells are observed during transformation from radial glia to astroglia *in vivo* [15]–[17]. These experimental results provide a simple means for the acquisition of sizeable quantities of immature stem cells from the de-differentiation of mature cells. Stem and/or precursor cells exist within a distinct tissue structure referred to as the niche, which regulates their self-renewal and differentiation [18], [19]. As recently demonstrated, the bone marrow microenvironment has a lower oxygen concentration than other tissues, and stem cells are localized within the hypoxic regions [20], thereby indicating that hypoxia may be crucial for the maintenance of stem cells. Under hypoxic conditions, the differentiation of embryonic stem cells, as well as precursor cells, is inhibited [21]–[23]. Conversely, the pro-differentiation gene is also downregulated as a result of *HIF1α* activation [24]. In this study, human adipose tissue stromal cells (ATSC) changed into more primitive stem cells after exposure to low oxygen and the use of a novel antioxidant for cell de-aging. We hypothesized that the antioxidant, 4-(3,4-dihydroxy-phenyl)-derivative (DHP-d), purified from *Phellinus linteus*, a powerful ROS scavenger that induces de-aging, can induce cell de-differentiation via increases in the potential for proliferation and differentiation of adult stem cells or ATSC. On the basis of these findings, we propose that a combined hypoxia/DHP-d system may improve an essential function in the maintenance of stem and/or precursor cells. We determined that differentiated adipocytes with proliferation potential can be compelled to de-differentiate. Recent observations have demonstrated that adult somatic stem cells have the capacity to participate in the regeneration of different tissues [25], [26], thereby suggesting that restrictions on differentiation are not completely irreversible and can be reprogrammed with de-differentiation and trans-differentiation processes.

In an effort to determine the characteristics of de-differentiation, we previously developed a well-defined biological system involving human ATSC with the potential to differentiate into multiple cell lineages, including neurons with active migration activity [26]. This study indicates that de-differentiation-induced ATSC can express molecular and biological characteristics distinct from native ATSC. After the induction of de-differentiation, the lifespan of the ATSC was increased significantly, and the therapeutic efficacies were improved in injured spinal cord and diabetes animals. The improved lifespan and differentiation ability of the de-differentiated ATSC strongly indicates that de-ATSC represent a highly useful candidate cell source for tissue regeneration and engineering for therapeutic approaches.

## Results

### Hypoxia/DHP-d Exposure in ATSC As Evidenced by Various De-Differentiation Behaviors via the Expression of Stemness Genes

**Figure 1:**
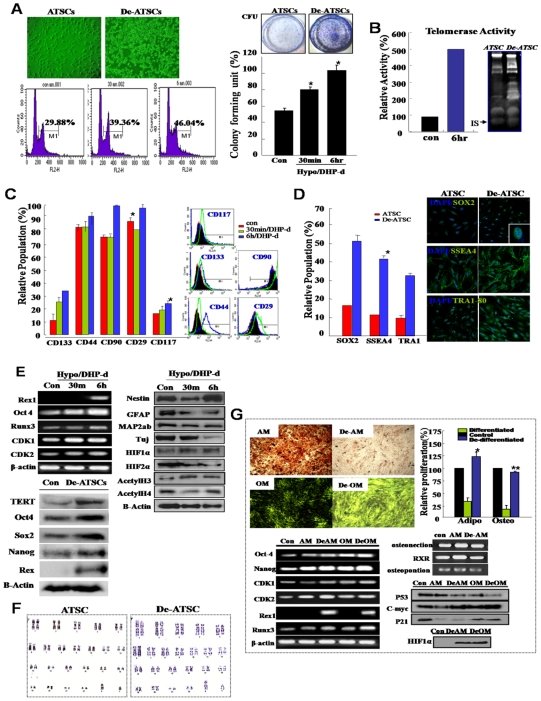
Combinational hypoxia/DHP-d induced various de-differentiation behaviors in hATSC cells. (A) The proliferation activity of cultured hypoxia/DHP-d-induced ATSC. Flow cytometric analysis and measuring colony forming units (CFU). For flow cytometric analysis, cells were cultured in 100-mm dishes at densities that ensured exponential growth at the time of harvesting. Harvesting and processing protocols were used to detect DNA via flow cytometry with propidium iodide. The percentages of cells in the G0/G1, S, and G2/M phases of the cell cycle were determined using a DNA histogram fitting program. Clonogenic cell growth experiments were conducted for the detection of colony forming units (CFU). (B) Telomerase assay of de-ATSC compared to control ATSC. Telomerase activity was assessed using a modified TRAP assay using synthetic oligonucleotide, telomerase-specific primer. (C) The expression of surface epitopes changed in de-ATSC. For phenotypic characterization by flow cytometry, de-differentiated ATSC and cultured ATSC adherent cells were incubated with antibodies. The labeled cells were analyzed with a FACScan argon laser cytometer. (D) Analysis of stem cells phenotypic change in cultured de-ATSCs through TRA 1-80, SSEA4, and Sox2 expression analysis. (E) Determination of differential expression of stemness, neural markers, and cell proliferation-associated genes by real time RT-PCR and Western blotting. (F) Detection and confirmation of the chromosomal normality of cultured de-ATSC via karyotyping analysis. (G) De-differentiation of terminal differentiated ATSC-derived bone and evaluation of fat and their pluripotencies through differentiated fat and bone staining and related gene expression analysis. Datas presented are presented as mean ±SD; *n>3*. * p % 0.05 and ** p % 0.01, Student's t test.

During prolonged culture periods in 10% FBS containing α-MEM medium, the population of control ATSC underwent a progressive reduction in proliferation potential, and finally underwent senescence after passage 13–15 (40–50 days in culture) (Fig. S1). The cell growth attenuation and cell death by senescence was highly involved in ROS (Reactive oxygen species) generation after extended passage through activation of apoptotic cell death signal molecules such as P38 and MAPK [27]. As shown in Fig. S1, in an experimental hypoxic and DHP-d-induced ROS scavenging environment, de-ATSC grew continuously for more than 3 months (>20 passages) and their cell cycle controlling factors such as CDK1, CDK2, and RUNX3 expression was prominently increased along with active growth activity compared to in the case of hypoxic or DHP-d single treatment (Fig. S2A). Moreover, hypoxic and DHP-d-induced de-ATSC showed a 2-fold increased colony forming unit (CFU) and increased synthetic DNA and over two-fold increased telomerase activity (Figure 1A, 1B, Fig. S2B). As following our experimental results, DHP-d-inducing cell proliferation activation phenotype was not derived from their protective function against hypoxia-mediated apoptotic cell death at the point of cell senescence (Fig. S2A). During extended cells subculture, we didn't identified apoptotic cell death signal such as Caspase 3, PARP, and Cytochrome C expression or actiation (Fig. S2A).

**Figure 2:**
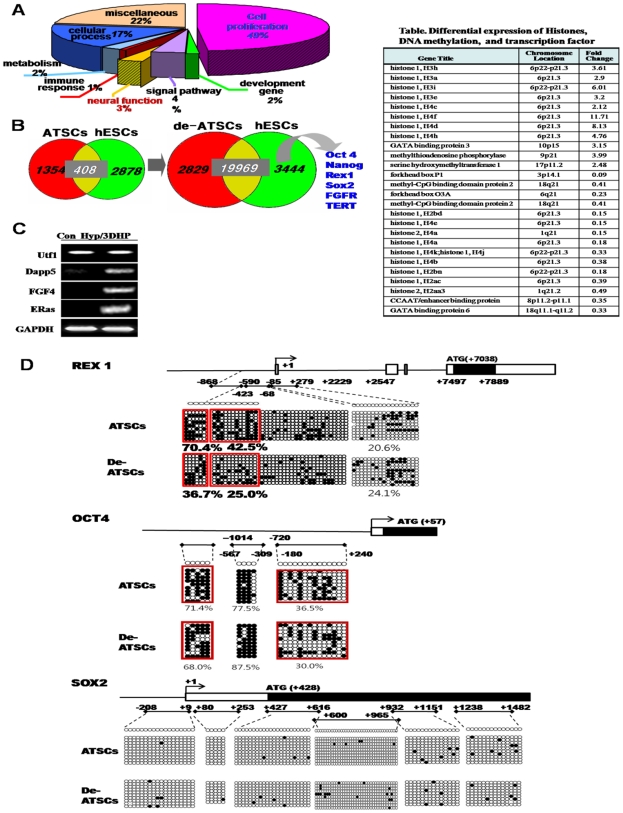
Functional categorization of differentially expressed gene profile and epigenetic reprogramming of stemness genes in de-ATSC. (A) Functional categorization of genes in de-ATSC. (B) Commonly expressed genes between the de-ATSC and hESC. Also, many kinds of histones and DNA methylation-related transcription factors and enzymes are overexpressed after ATSC de-differentiation (Table). (C) Differential embryonic genes, Utf1, Dapp5, FGF4, and Eras expressions in de-ATSCs. (D) Evaluation of epigenetic modifications through methylation analysis on promoter regions through bisulfite modification and sequencing of genomic DNA. The related method was explained in Materials and Methods S1.

The phenotypic characteristics of the de-ATSC showed significantly increased CD90, CD29, CD44, CD117, and CD133 surface epitope-harboring populations and also they appeared gradually increased embryonic stem cells markers, such as Sox2, SSEA4, and TRA1-80 in the results of FACS and immunocytochemical analysis (Figure 1C and 1D). Low oxygen/DHP-d was determined to exert prominent effects on the overexpression of a variety of proliferation-associated genes, including *RUNX3, CDK2, Cyclin D2, CDK1*, and telomere reverse transcriptase (TERT; Figure 1E). As shown in Figure 1E, after 3 days of *in vitro* culture, the de-ATSC overexpressed several stemness genes such as Oct4, sox2, Nanog, and Rex1 with downregulation of the mature neural marker proteins, GFAP, TuJ, and MAP2ab. As following western blotting and FACS analysis, the de-ATSC showed extended cell growth through the activation of JAK/STAT3 and ERK1/2 and overexpression of c-myc protein and a high ratio of S phase in cell cycles (Figure 1A). In one essential test conducted to determine whether low oxygen/DHP-d induced the expression of early developmental genes in cultured ATSC, we evaluated the expression of *Oct-4 (POU5F1), Sox-2, Rex-1*, *MMP2*, *TERT*, *Utf1*, *Dapp5*, *FGF4*, *ERas*, and *Nanog* genes (Figure 1D, Figure 2). Following 6 hours of exposure to low oxygen/DHP-d, human ATSC expressed Oct-4. Most of the target genes of Oct4 were also upregulated, including Rex1, Nanog, and Sox2, in addition to Nestin with downregulation of mature lineage markers such as MAP2ab and GFAP (Figure 1E). Our study has also provided some additional observations regarding nuclear remodeling, including the acetylation and demethylation of histone H3 (Figure 1E). As following cytogenetic analysis and single nucleotide polymorphism (SNP) experiment of de-ATSCs, our de-differentiation techniques did not induce chromosomal abbreviations (Figure 1F) or point mutations (Fig. S4). On the other hand, we induced the de-differentiation of fully differentiated fat and bone cells using a hypoxia/DHP-d system. As a result, the fully differentiated cells exhibited de-differentiated stem cell behaviors, including active growth with CDK1, CDK2, RUNX3, and the upregulation of several stemness genes, Rex1 along with the definitive downregulation of mature differentiation markers (Figure 1G). The bone and fat cells staining and related genes expression pattern revealed that hypoxia/DHP-d system also effectively induces the de-differentiation of fully differentiated, mature bone and fat cells (Figure 1G) and also hypoxia/DHP-d induced de-ATSCs showed more effective chondrogenesis and osteogenesis than those of control ATSCs (Fig. S3B).

### Hypoxia/DHP-d Effectively Induced Epigenetic Reprogramming on the Promoter Regions of Stemness Genes

The analysis of gene expression levels indicated that <4% of the total genes were expressed at greater than 2.2-fold for different levels in ATSC and de-ATSC, as indicated by the *r* values ( = 0.67). A comparison of the expression of those showed that cell proliferation-associated genes were profoundly upregulated in de-ATSC (49%). Common gene expression in ATSC and human embryonic stem cells (hES cells) (n = 408) showed dramatically increased levels of commonly expressed genes in the de-ATSC and hES cells (n = 19969) including stemness genes such as Oct4, Sox2, Nanog, Klf4, FGFR, and TERT. Genes associated with growth involving the signal pathway including JAK/STAT3 were also prominently activated or overexpressed (Figure 2A, 2B). Furthermore, we determined that chromatin remodeling, epigenetic reprogramming-related genes, and development related genes were also overexpressed by >2-fold in de-ATSC (Figure 2C, Table within Figure 2). In an effort to determine whether hypoxia/DHP-d conditions were capable of eliciting epigenetic modifications on exogenous chromatin templates, we evaluated changes in DNA methylation in the stemness genes promoter regions. We also conducted a bisulfate sequencing analysis in order to establish the 5′-3′ CpG methylation profiles across each test gene proximal promoter, the proximal enhancer, and the early transcription start site (TSS). In the case of Rex1, 6 amplicons were assessed, collectively converting the potentially methylated CpG dinucleotides within nucleotides -869 to +7889 relative to the TSS (Figure 2D). Seven regions were also analyzed in the Oct4 promoter, encompassing the CpGs within nucleotides -2995 to +240 relative to the TSS (Figure 2D). The proximal Sox2 region and the TSS region assessed did not significantly alter the methylation (Figure 2D). The Rex1 region assessed was highly methylated in the ATSCs control and was meaningfully demethylated in the second region from 70.4 (ATSC) to 36.7% (de-ATSC) and the 3^rd^ region from 42.5 to 25% (Figure 2D). This Oct4 methylation pattern was more or less downregulated in the de-ATSC (5^th^ region, 71.4%, 7^th^ region, 36.5%) as compared to the control ATSC (5^th^ region, 68.0%; 7^th^ region, 30%; Figure 2D).

### Low Oxygen/DHP-d-Induced ATSC De-Differentiation with JAK/STAT3 and MAPKinase Activation and Rex1 and Oct4 Upregulations

**Figure 3:**
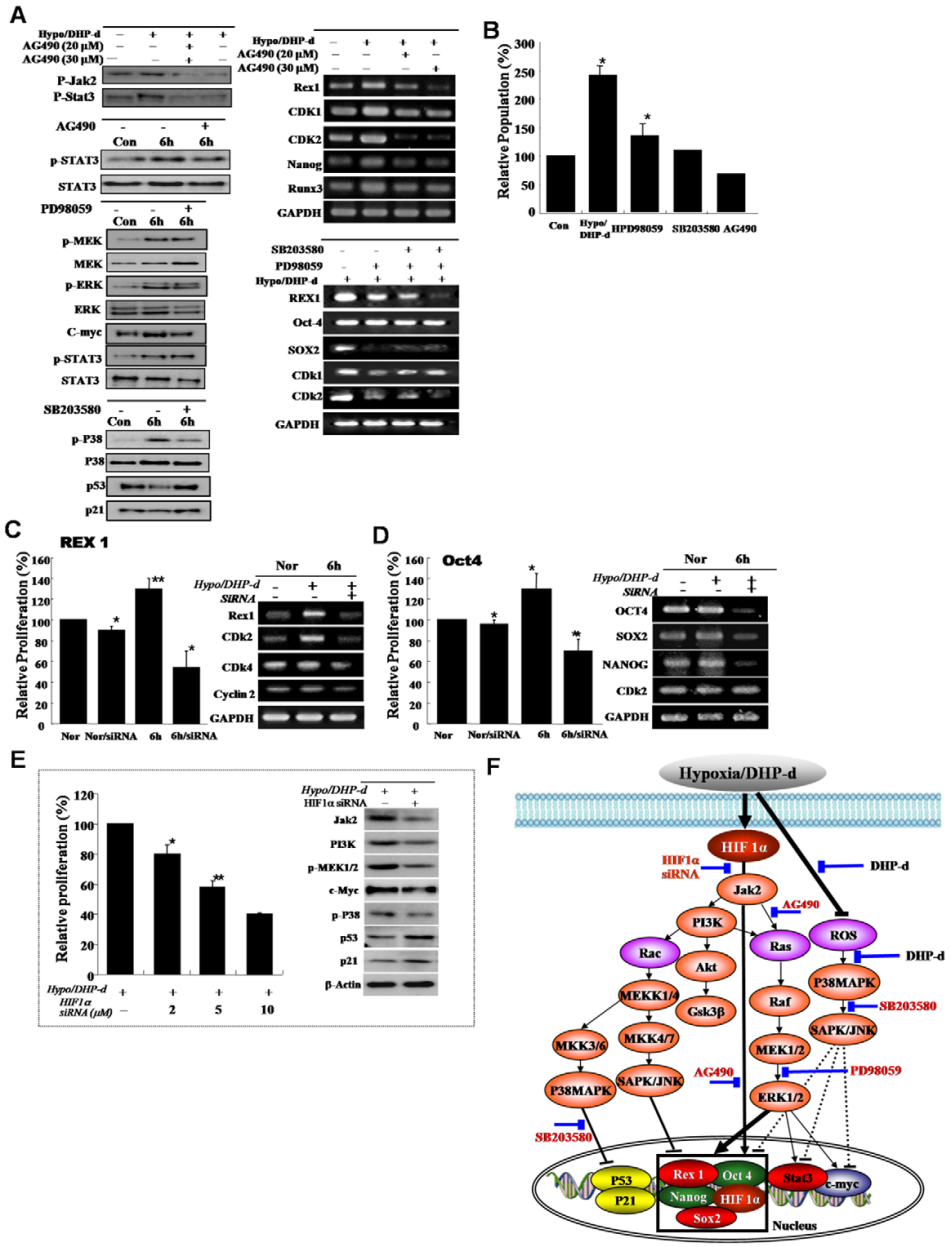
Differential expression of the growth-related signal proteins and Rex-1 involvement in active growth of low oxygen/DHP-d exposed ATSC. (A, B) The involvement of the JAK/STAT3 and MEK signal protein in active cell growth after de-differentiation of ATSC. For the confirmation of differentially expressed proteins following the de-ATSC, the cells lysates were subjected to SDS-PAGE analysis and transferred to nitrocellulose membranes. Optimally diluted antibodies were incubated with the membranes. The relative band intensities were determined using Quality-one 1-D Analysis software. (C) Prominent inhibition of de-ATSC growth by Rex1 siRNA and (D) Oct4 siRNA. Two complementary hairpin siRNA template oligonucleotides harboring the 21 nt target sequences of the human Rex1 were employed for transient transfection. Three separate Rex1 siRNAs and scrambled siRNAs with the same nucleotide content were assessed. For the detection of the inhibition of de-ATSC growth, we transfected Rex1 siRNA into de-ATSC counted dye-exclusive viable cells for 6 days. (E) Functional involvement of HIFs in de-ATSC proliferation, proliferation controlling signal protein, and stemness genes expression. (F) Schematic flow chart of the low oxygen/DHP-d induced ATSC proliferation signal pathway involving Rex1, Nanog, p53, p21, and c-myc gene expression and activation in the nucleus. Datas presented are presented as mean ±SD; *n>5*. * p % 0.05, and ** p % 0.01, Student's t test.

In an effort to identify the possible activated signaling molecules involved in active cell proliferation following hypoxia/DHP-d exposure, the total protein levels and phosphorylation status of ERK 1/2 was assessed in the hypoxia/DHP-d exposed ATSC. ERK1/2 phosphorylation was clearly upregulated 3 hours (*P< = *0.05) after hypoxia/DHP-d exposure. The phosphorylation status reached the maximum at 3 hours and then was reduced to an undetectable level at the subsequent time points (Figure 3A). Coincident with the hypoxia/DHP-d-induced ATSC proliferation, phosphorylated *Akt* was activated weakly in the de-ATSC and was markedly increased 6 hours after exposure to a hypoxic environment (Figure 3A, 3B). Further, de-ATSC proliferation was also mediated by *JAK/STAT3* phosphorylation along with Rex-1, CDK1, CDK2, and RUNX3 expression (Figure 3B). When treated with JAKinase inhibitors, STAT3 phosphorylation and associated proliferation factors involving cell growth and CDK1, CDK2, Rex1, Nanog, and RUNX3 expression was decreased profoundly (Figure 3A and 3B, 3C). As is shown in Figure 3A, the phosphorylated Akt was significantly upregulated after 6 hours of exposure to low oxygen/DHP-d. Hypoxia/DHP-d-induced ATSC prominently activated PI3K, GSK3β, MEK, MEKK, and raf proteins during cell proliferation, and also induced a profound reduction in ERK1/2 and p38 activation and CDKs, Rex-1, and Sox-2 downregulation following treatment with specific inhibitors. (Figure 3A, 3B). The activation of ERK1/2 and Akt in de-ATSC via hypoxia/DHP-d resulted in the induction of stemness transcription factor expression, and in particular, Rex1 expression (Figure 3A). For further study of the roles of Rex1 in the proliferation of de-ATSC, the ATSC were transfected with Rex1 silencing siRNA prior to and after exposure to low oxygen tension and DHP-d. As is shown in Figure 3C, the Rex1 siRNA transfected de-ATSC profoundly inhibited cell growth and Rex1 gene expression. The attenuation of cell proliferation by Rex1 inhibition was attended by the downregulation of CDK2, CDK4, and Cyclin 2 (Figure 3C). Collectively, hypoxia/DHP-d-induced Rex-1 expression was shown to be positively regulated by JAK/STAT3 and p38/MEK phosphorylation, and Rex-1 controlling hypoxia/DHP-d-induced cell proliferation and dedifferentiation (Figure 3). As following Oct4 knockdown experiment, Oct4 was also actively involving in cell proliferation and stemnesses genes expression after dedifferentiation induction (Figure 3D). After hypoxic stimuli, the expression of HIF1α was induced and their siRNA transfection resulted in attenuation of hypoxic/DHP-d activated stem cell growth and related signal protein activation, including MAPK, JAK/STAT, and AKT (Figure 3E). Also, HIF1α siRNA transfection prominently induced downregulated stemness genes expression, such as Rex1, Sox2, Oct4, and Klf4 with prominent growth attenuation (Figure 3E, 3F).

### De-Differentiated ATSC Showed p38/JUNK-Mediated Active Migration and Improved Differentiation Potencies *In Vitro* and *In Vivo*

**Figure 4:**
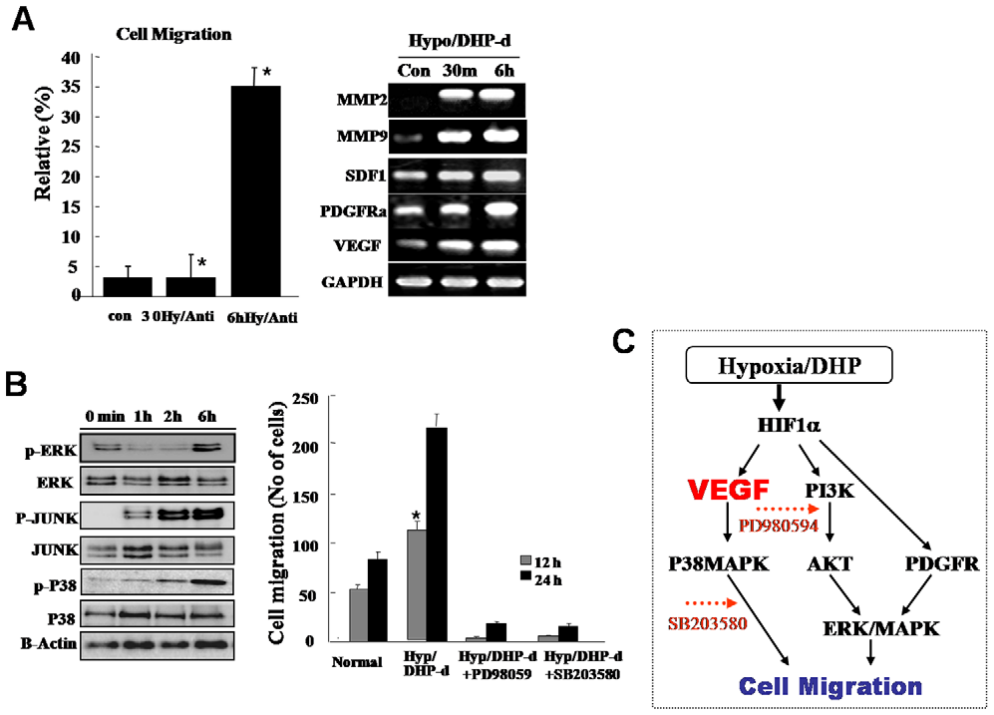
De-differentiated ATSCs evidenced active cell migration. (A) Migration activity of de-ATSC was evaluated as a percentage of the spontaneous migration and related functional factors. The migration activity of the dedifferentiated ATSC *in vitro*, the cells were transferred to culture dishes containing low serum growth medium. The cultured cells were transferred into transwell membranes (8 µm pore size), coated on both sides with laminin. In the upper chamber, both of cells were preincubated in a CO_2_ incubator. For analysis, migrating cells on the lower surface were air-dried and counterstained with Harris hematoxylin and the numbers of cells on the lower surfaces were assessed. Ten x20 fields per insert were counted. (B) The migration activity of dedifferentiation of ATSCs was caused by ERK, JUNK, and P38 phosporylations. (C) Proposed molecular mechanism of cell migration after ATSCs reprogramming by DHP-d/Hypoxia. Datas presented are presented as mean ±SD; *n>4*. * p % 0.05, and ** p % 0.01, Student's t test.

**Figure 5:**
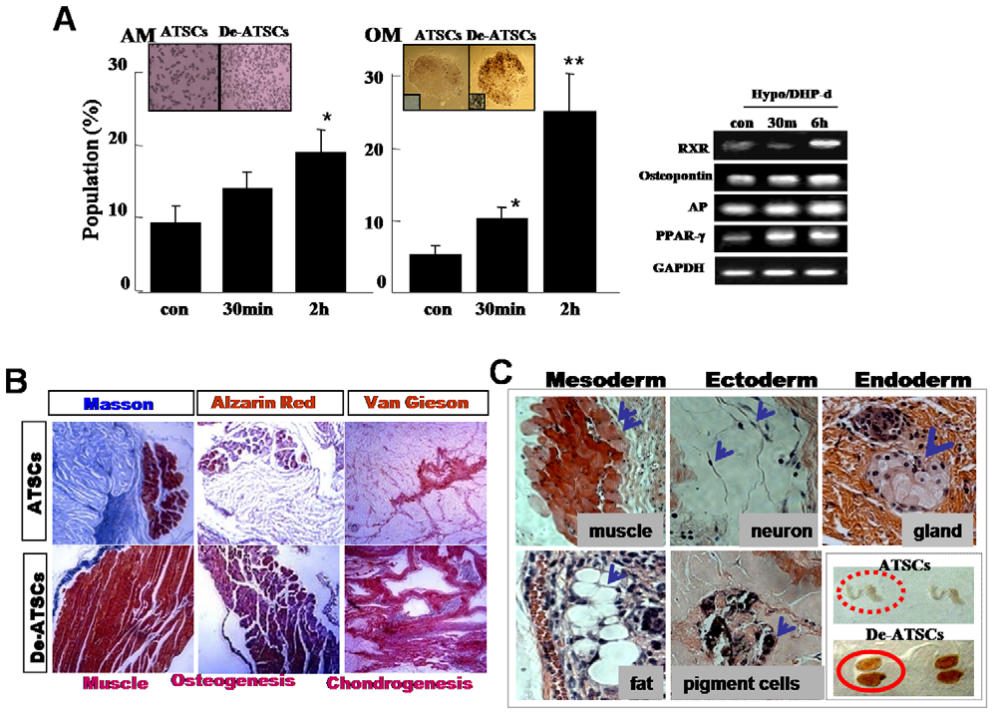
Determination of adipogenic, osteogenic, and muscle differentiation potencies of de-ATSC. *In vitro (A)* and *in vivo* Determination of adipogenic, osteogenic, and muscle differentiation potencies of de-ATSC SCID/NOD mice (*B*). The transplants were recovered 6 weeks later, stained via Alzarin Red (bone), Masson (muscle, chondrocyte), and Van Gieson (chondrocyte) and collagen IV immunostaining. (C) Teratoma formation from engrafted de-ATSCs in SCID mouse and development of three germ layer-derived tissue or organ such as muscle, neuron, pigment cell, and gland in teratoma after subcutaneously implantation in 8-week-old immunodeficient beige mice. The paraffin-embedded sections were stained via H&E, Alzarin Red (bone), Masson (muscle and chondrocytes), and van Gieson (chondrocytes). Datas presented are presented as mean ±SD; *n>4*. * p % 0.05, and ** p % 0.01, Student's t test.

**Figure 6:**
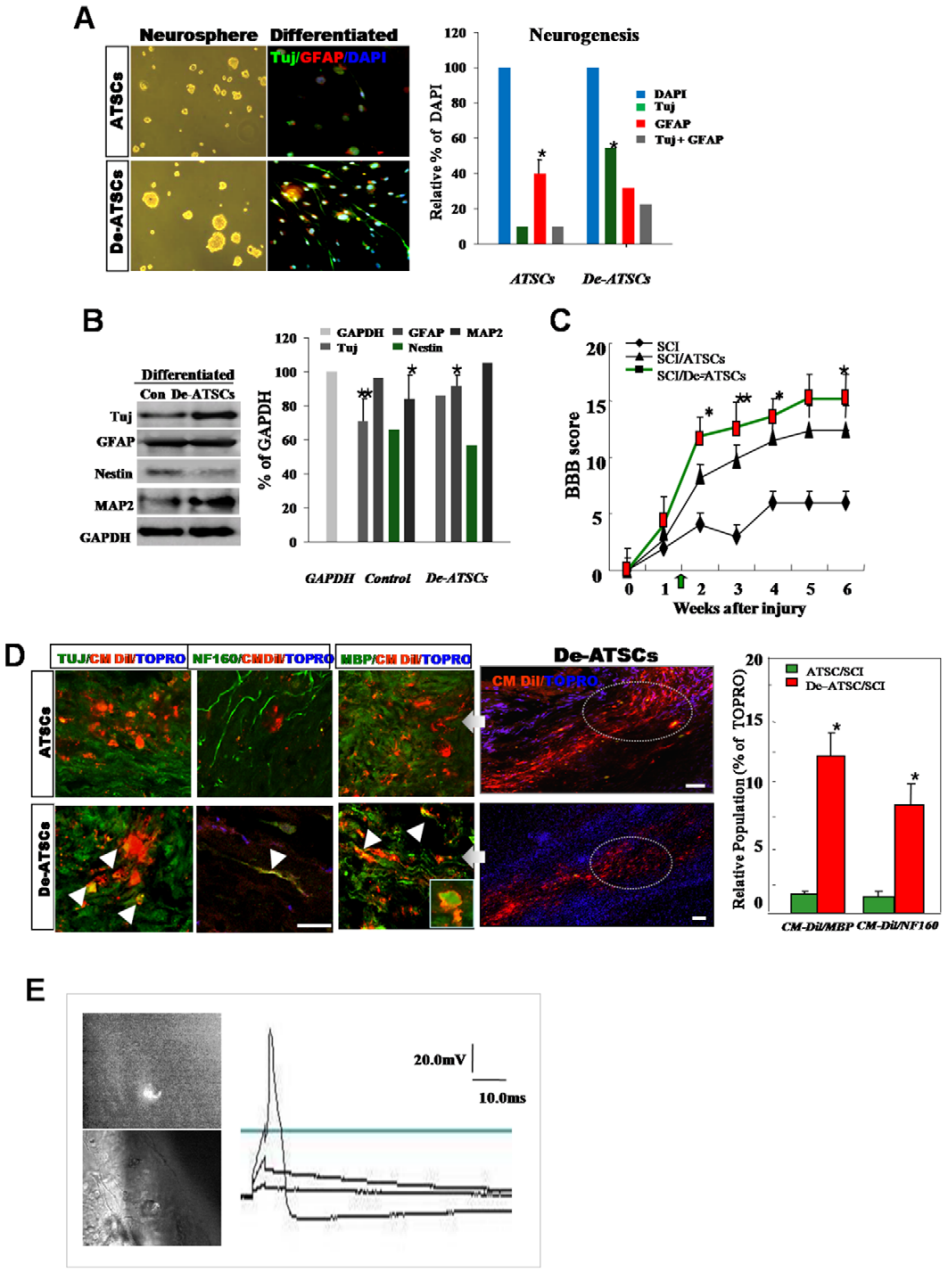
The regenerative potency of de-ATSC and their functional efficacy in spinal cord injury rat model. (A)(B) Evaluation of the neurogenic potency of de-ATSCs *in vitro*. Neurospheres were cultured and attached on PDL-laminin double-coated well plates. For analysis of the neural markers expression, fixed cells were incubated with primary antibodies against anti-TuJ 1 and anti-GFAP and fluorescence conjugated secondary antibodies sequentially. We then analyzed the cells via fluorescence microscopy. And also we confirmed the neurogenic potency of differentiated de-ATSC by immunoblotting and their quantitative evaluation. (C) In order to determine whether de-ATSCs evidence a regenerative effect *in vivo*, we compared motor function using a modified BBB hind limb locomotor rating scale 10 days after SCI. (D) And also we evaluated transdifferentiation potency of engrafted cells by immunohistochemical analysis of spinal cord tissue. Detail experimental processes was explained in Materials and Methods. High efficiency of trans-differentiation ability into the neuron and myelin in lesion site of spinal cord. Rounded, dot circle showed specific region for immunohistochemical analysis of regenerative activity of de-ATSCs in SCI tissue. Raw data from each experiment were analyzed via analysis of variance with Fisher's or t-tests. Scale bars represent 40 *μ*m. (E) Functionally active, transdifferentiated neurons from engrafted cells was evaluated by recording of their evoked action potential before, immediately after and 30 days after the sciatic axotomy. Bipolar hooked platinum recording and stimulating electrodes were used to induce and record electrical activity. The evoked action potential in responding to the stimuli (one ms, 20.0 mV) in the ipsilateral sciatic nerve was recorded. Datas presented are presented as mean ±SD; *n>5*. * p % 0.05, and ** p % 0.01, Student's t test.

One of the most important events in wound repair is the migration of surrounding epithelial cells into the wounded lesion site. De-ATSC had excellent migration and wound healing activities against mechanical scratch-induced damage (Figure 4A). Basically, de-ATSC overexpressing VEGF and PDGFRα function as paracrine growth factors, and induced active cell migration prominently involving the phosphorylation of MAPK cascade proteins, such as p38, ERK1/2, and JUNK (Figure 4). In particular, the migration of scratched de-ATSC was blocked to a significant degree by the inhibition of p38 and pERK1/2 phosphorylation by SB203580 and PD98059 (Figure 4B). ATSC have been identified as progenitors of skeletal tissues, and differentiate into osteoblast-like cells in cultures supplemented with ascorbic acid and a glucocorticoid source. ATSC typically begin to accumulate calcium and lipid droplets following 2–4 weeks of induction in osteogenic and adipogenic differentiation media. However, de-ATSC was shown to accumulate significant quantities of calcium and lipid droplets and the differences in the efficiency of nodule and lipid droplet formation between the naive and de-ATSC. As is shown in Figure 5A, up to three times as many lipid droplets and nodules were detected in the de-ATSC as compared to the control ATSC. After culturing of de-ATSC (passage 5) in osteogenic differentiation media, we conducted von Kossa staining for calcium deposits. These results are generally consistent with what has been observed in conjunction with the overexpression of adipogenesis- and osteogenesis-related transcription factors, including RXR, osteonectin, AP, and PPAR-gamma, after de-differentiation (Figure 5A). That also appeared highly enhanced bone and muscle formation by de-ATSC after Alzarin Red, and Masson staining of the implant tissue sections from de-ATSC engrafted SCID mice (Figure 5B). Specifically, collagen IV was highly expressed in the de-ATSC engrafted in SCID mice (Figure 5B). Moreover, five weeks after the transplantation of de-ATSC in SCID mice, the results of the hematoxylin-eosin, three germ layer-derived several organs were generated inside of small teratoma (Figure 5C). Compare to control ATSC cells, we observed extremely upregulated TuJ and MAP2ab and low levels of Nestin expression in the de-ATSCs after neural differentiation (Figure 6A). A large population of differentiated de-ATSC showed morphologic and phenotypic characteristics of astrocytes (GFAP), and neurons (MAP2ab and NF160 [approximately 45% of the total population]; Figure 6A). Our western blotting results also confirmed that more efficient transdifferentiation potency of de-ATSCs than that of control ATSCs (Figure 6B).

### Effected Regenerative Behavior of De-ATSC in Injured Rat Spinal Cords

In order to determine if de-ATSC have regenerative and therapeutic effects in an injured spinal cord rat model, we evaluated motor functions using a modified Basso, Beattie, and Bresnahan (BBB) hind limb locomotor rating scale. As shown in Figure 5b, all animals subjected to spinal cord injury (SCI) had extensive deficits in hind limb function over the first few days post-injury as compared to the uninjured animals, suggesting that all animals experienced a similar degree of SCI. By 3 weeks post-SCI, rats administered the de-ATSC and the ATSC controlled consistently supported their weight during planar stepping and had a predominantly rotated paw position during locomotion. During the same time period, the injured animals injected only with matrigel had limited joint locomotion (Figure 6C). Although functional efficacy was observed in both of the cell-engrafted SCI rats, the results of behavioral analysis showed that the locomotor function and regeneration efficacy of de-ATSC-engrafted SCI rats were restored more significantly, with higher levels of regenerative activity than those of control ATSC-engrafted rats (Figure 6D, 6E). A higher percentage (12.5% [de-ATSC] and 8.5% [ATSC]) of motor neuron and MBP-positive myelin differentiation (NF160/CM-Dil double positive) was detected in de-ATSC (2.4 and 3.8% of each engrafted cells) of neural and MBP-positive myelin differentiation in the lesion sites of SCI (Figure 6D). Engrafted de-ATSCs appeared improved transdifferentiation potency into electrophysiological active motor neuron in lesion site of injured spinal cord (46%; about 7 action potential-positive cells/15 transdifferentiated de-ATSCs into neuron in lesion) (Figure 6E). In contrast of de-ATSCs, control-ATSCs never showed action potential carrying, transdifferentiated neuron in lesion site of spinal cord.

### Improved Functional Efficacy of De-ATSCs for Diabetes Therapy

**Figure 7:**
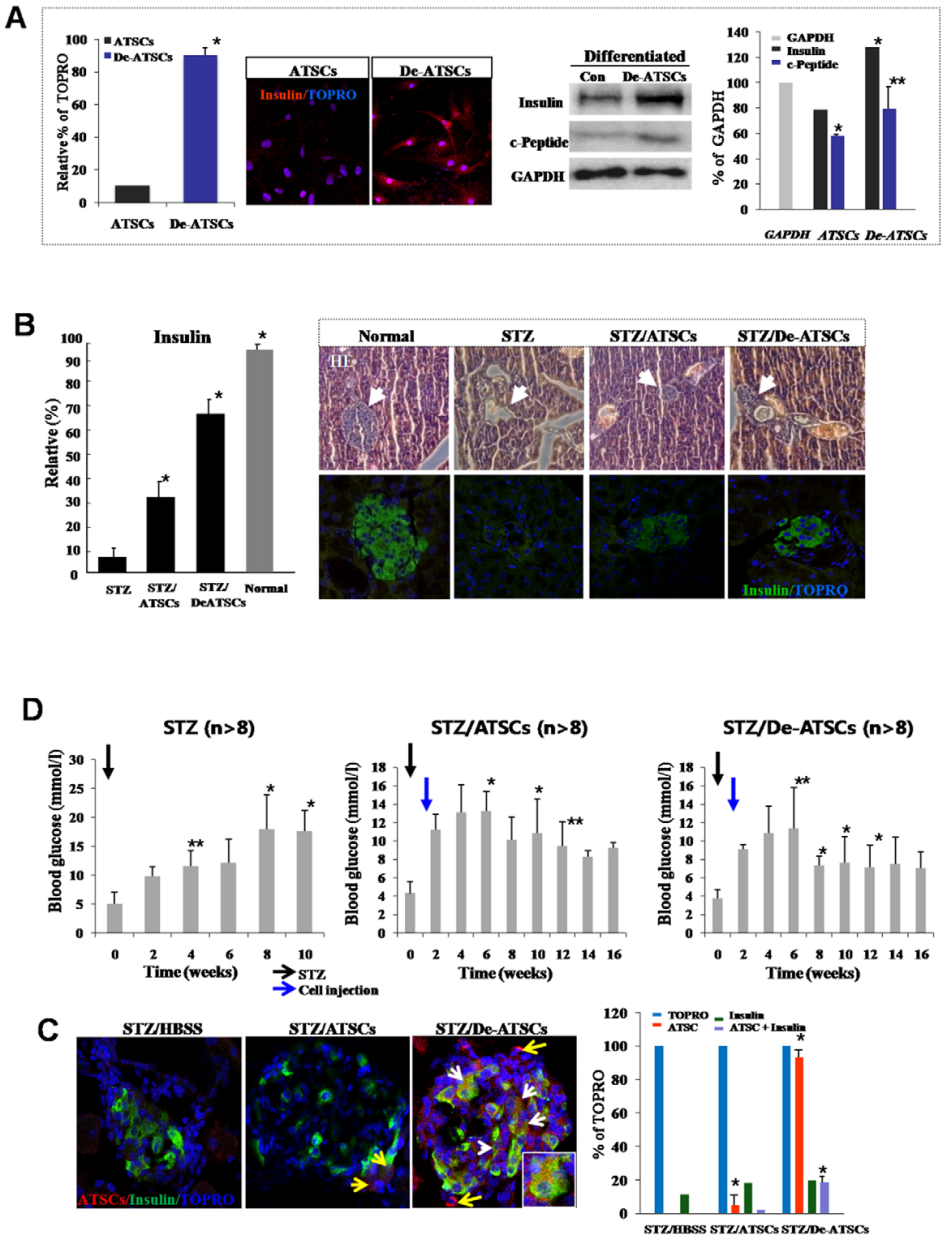
Reversion of the hyperglycemic condition and regeneration of beta-pancreatic islets in de-ATSCs-treated type 1 diabetic mice. (A) Beta cell differentiation potency of de-ATSCs cells. For beta-like cell differentiation, cells were cultured in “N2 media+NA” containing DMEM/F12. After the induction of differentiation, we conducted immnocytochemistry and western blotting using and insulin and c-peptide antibodies. (B) Quantitative measurement of secreting insulin from de-ATSCs engrafted type1 diabetic animals (left). For analysis of pancreatic islets tissue-derived insulin secreting functions after anti-insulin/green fluorescence marked ATSCs control and de-ATSC cells treatment in diabetes animals (right). Pancreatic islets dissected on day 21 from control and STZ-treated mice were stained with hematoxylin and eosin or (C) immunostained for mouse insulin. Scale bars  = 20 *μ*m. White arrows revealed that transdifferentiated and insulin secreting engrafted de-ATSCs. (D) Measurement of blood glucose concentration before and after each cell (2×10^5^ cells) engraftment. Pancreatic damage was induced with intraperitoneal injection of 50 mg/kg of body weight STZ daily for five consecutive days. Datas presented are presented as mean ±SD; *n>*8. Black and blue arrows appear the date of STZ and cells injection each other. Detail experimental processes was explained in Materials and Methods. Data are represented as mean ±SD; *n>8*. * p % 0.05, and ** p % 0.01, Student's t test.

For evaluate transdifferentiation and regenerative activity of de-ATSC cell into endodermal lineage of cell, we induce beta cell differentiation of control and de-ATSCs *in vitro* and chemical-induced *in vivo* diabetes animal model. At result, de-ATSC cell was prominently trans-differentiated into endoderm-originated beta cells after induction of differentiation. Differentiated de-ATSC secreted a considerable amount of insulin. However, insulin producing cells was very rare in the case of control ATSCs after differentiation induction (Figure 7A). Immunohistochemical analysis of insulin-positive cells in pancreatic sections also proved more effective de-ATSCs treatment of diabetes compare to control ATSCs, since mass of insulin-producing cells became comparable to that of healthy controls (Figures 7B). As following tissue immunohistochemical study, in the case of de-ATSCs engrafted STZ-treated animals showed almost 67% of damaged islet cells was recovered and they secreting insulin but control ATSCs engrafted animal showed more or less low density of islet cells (32%) was showed insulin secretion (Figure 7A, 7B). Engrafted de-ATSCs also showed effectively transdifferentiated into insulin secreting beta cell but we never found beta cell transdifferentiated ATSCs control cells *in vivo* diabetes animals (Figure 7C). Insulin-positive cells did not appear in other organs, such as liver, spleen, lung, and bone marrow (data not shown). To determine whether or not de-ATSCs contribute to the repair of pancreatic functions, we injected de-ATSCs or ATSCs control intravenously into STZ-treated animals. After injection of cells, blood glucose level of diabetic animals more rapidly returned to normal levels when they received de-ATSC than that of control ATSCs on day 6–8 (Figure 7D) and engrafted de-ATSCs cells was efficiently transdifferentiated into insulin secreting beta cells (Figure 7C). Therefore, de-ATSCs effectively acted in concert in therapy of experimentally induced diabetes.

## Discussion

The results of several previous experiments have shown that even completely differentiated cells can de-differentiate into precursor cells capable of acquiring different structures and functions. In our study, de- ATSC overexpressed not only Oct-4, Sox-2, Nanog, and Rex-1, but also c-Myc for the acquisition of active self-renewal activity with pluripotency. On the other hand, de-ATSC exhibited prominent p21 and p53 gene downregulation. Our results show that ATSC can undergo an increase in developmental potential following reprogramming via the overexpression of the embryonic transcription factor, Rex1, Oct4, and Oct4-dependent Nanog and Sox2. Most notably, de-ATSC reprogrammed somatic nuclei to express the POU family member homeodomain transcription factor genes, Oct 4 and Rex 1, via a process necessitating DNA demethylation. Thus, the components of pluripotent ATSC cells have the potential to elicit reprogramming events in a somatic genome. The proliferation of de-ATSC is promoted significantly by exposure to hypoxia/DHP-d with highly improved pluripotency. The results of these studies indicate that ATSCs possess their own multipotency to de-differentiate into more primitive stem cells, with the exception of chromosomal abnormalities and point mutations. Thus, the exposure of ATSC to low oxygen/DHP-d may provide a good *in vitro* model to explore the mechanisms of re-differentiation from the de-ATSC, which would provide insight into the molecular mechanisms of ATSC proliferation. Although, the ERK MAPKs generally regulate cell growth and differentiation, and the JNK and p38 family MAPKs preferentially mediate stress, there is now an increasing amount of evidence to suggest that the activation of the ERK MAPKs can also be stimulated by a variety of stress stimuli, including low oxygen tension [28], [29]. Hypoxia/DHP-d can activate ERK MAPKs via pathways independent or dependent [30] of Ras and Raf activation. Our results indicated that hypoxia and DHP-d can activate MEK and ERK1/2 within a few days of de-differentiation induction. Such a change was also detected with respect to Akt activation. This study showed, for the first time, that low oxygen/DHP-d could induce a reversible change of the ATSC to a more immature de-differentiated state, via not only the PI3K/Akt-mediated pathway, but also via JAK/STAT3-mediated signals. The proliferation-associated molecular signal pathway with a high level of TERT activity occurring in the de-ATSC and the pattern of gene expression revealed a reversion toward a more immature phenotype of the cells. The results provided some insight into the manner in which gene expression in human ATSC responds to hypoxia/DHP-d. After de-differentiation induction, HIF1α expression was increased. HIF1α knockdown induced stemness gene and growth signature downregulation that revealed that dedifferentiation induced HIF1α expression with cell growth and growth controlling stemness gene expression directly or indirectly [31]. The analysis of the differentially expressed genes indicated that the upregulated genes triggered by hypoxia/DHP-d can be clustered into different functional groups. Some genes have been shown to be responsible for cell growth, VEGF-involving angiogenesis. And also we never identified cell death-related signature. These results indicated that hypoxia/DHP-d induced the activation of ATSC and accelerated migration and proliferation via de-differentiation processes except apoptotic cell death stimuli. Among the upregulated genes, cell growth regulatory genes were often observed (49%), including cyclin E2, replication factor C, cyclin D1, replication protein A2, and cell division cycle associated 7. Some are relevant to neurogenesis, migration, and remodeling of ECM and are involved in the regulation of cellular responses to ECM, including MMP-2. Our results also indicated that hypoxia/DHP-d can activate MEK, p38, and ERK1/2 and those signal molecules effectively induced de-ATSC migration involving wound healing.

In our study, the low oxygen/DHP-d treatment of ATSC provides a simple method for the production of primitive stem cells via ROS controlled de-aging process, and may also be utilized in the investigation of the mechanisms underlying differentiation and de-differentiation. Based on the morphologic and immunocytochemical features observed herein, we showed that ATSC induced by hypoxia/DHP-d stimuli are de-differentiated, rejuvenated immature stem cells, and also de-ATSC have excellent multipotency for endodermal beta cell and ectodermal neuron differentiation. Specially, de-ATSC has dramatic regenerative ability in spinal cord injured rats and diabetes mice with improved motor function. Given the active growth and differentiation potency induced by the de-differentiation processes of adult stem cells and the relative ease with which genetically unchanged multipotent stem cells can be harvested. Finally, our ATSC reprogramming strategy may provide us with a potentially considerable reservoir of novel stem cells for use in novel and improved cell-based disease therapies.

## Materials and Methods

### Isolation and Culture of Adipose Tissue-Derived Stem Cells

Donor-derived the raw adipose tissues were processed in accordance with established methodologies in order to determine the stem vascular functions [23], [24]. Human raw fat tissue obtained from the patient abdomen (as following patient's approval document) was processed according to established methodologies to determine stem cell vascular function. In order to isolate the stem cells, the samples were digested with 0.075% collagenase IV (Sigma) and centrifuged at 1200×g for 10 minutes to acquire a high density cell pellet. The pellet was then suspended in red blood cell (RBC) lysis buffer (Biowhittaker, Walkersville, MD, USA) and incubated for 10 minutes at room temperature to lyse the contaminating RBCs. The stem cell pellet was then collected and incubated overnight at 37°C/5% CO_2_ in 10% FBS containing α-MEM medium (GIBCO BRL, CA, USA). This work was approved by Seoul National University Institutional Review Board (IRB No. 0603/001-002-07C1) and the ethics committee specifically approved that procedure.

### ATSC De-Differentiation Induction by Low Oxygen Tension and DHP-d

Human ATSC were cultured to passage 5–6 (P5-P6). ATSC were cultured at 37°C in a humidified atmosphere containing 5% CO_2_ with α-MEM containing 10% FBS and antibiotics (all obtained from Gibco BRL). In present study, we also used 4-(3, 4-Dihydroxy-phenyl) (DHP) derivative purified from *phellinus linteus*, a medicinal fungus known as "Sang-hwang" in Korea for cell reprogramming. For DHP derivative purification from phellinus linteu, DHP derivative was extracted with ethanol for 1 week. The ethanol extract was suspended in hexane and H_2_O. The hexane extract was subjected to repeated silica gel (0.063–0.200 mm, Merck) column chromatography, using a hexane-ethyl acetate gradient as the eluting solvent. High-performance liquid chromatography (YMC-Pack ODS-AM, 250×6.0 mm, ID 5 µm; with acetonitrile 70%, flow rate provided DHP derivative. For hypoxic stimulation, the culture plates were incubated in the chambers (Billups-Rothenberg, CA, USA) flushed with a gas mixture containing 1% O_2_, 5% CO_2_, and 94% N_2_ at 5–8 psi for 2 or 6 hours in the novel antioxidant (DHP-d [10 ng/ml])-containing culture medium. The humidified chambers were sealed and maintained at 37°C, then incubated further. The controls included parallel cultures in which the cells were exposed to normoxic conditions (21% oxygen tension). After de-differentiation, de-ATSC were maintained in a DHP-d (10 ng/ml)-containing culture medium. The media were initially replaced at 48 hours and then every fourth day thereafter. Purified and characterized DHP-d was kindly supplied by Dr. Jeon (Korean Research Institute of Biological Biotechnology).

### Non-Radioisotopic Telomerase Assay

Telomerase activity was assessed using a modified telomeric repeat amplification protocol (TRAP) assay, in accordance with the manufacturer's instructions (BD Science). Protein extracts were prepared from the ATSC controls and the de-ATSC. Protein extracts (0.5 µg) prepared from each cell line was incubated in the presence of synthetic oligonucleotide (telomerase-specific primer, 5′-AATCCGTCGAGCAGAG TT-3′), which could be the substrate for the addition of telomeric repeats by telomerase. If telomerase activity was detected in the extracts, the oligonucleotide was elongated and could function as a template in subsequent polymerase chain reactions (PCR). PCR was conducted in the presence of nucleotides, and the formation of the amplification products was assessed via the monitoring of telomerase repeat amplification. PCR reaction products were separated on 12.5% non-denaturing acrylamide gels and stained using Syber-Gold dye (Molecular Probes, Eugene, OR, USA). Quantification of telomerase for comparisons with telomerase activity in the de-ATSC and the ATSC controls was conducted via the PCR enzyme-linked immunosorbent assay procedure suggested by the manufacturer (BD Science).

### Oligonucleotide Microarray and Data Analysis

Samples for gene array analysis were prepared from the total RNA, and microarray analysis was conducted in accordance with the manufacturer's recommendations. Fragmented cRNA (15 mg) was hybridized for 16 hours at 45°C to the HG-U95A array for the comparison study (Affymetrix, Santa Clara, CA, USA). After hybridization, the probe arrays were scanned at 3 mm resolution using the Genechip System confocal scanner made for Affymetrix by Agilent. Affymetrix Microarray Suite 4 was used to scan and analyze the relative abundance of each gene as derived from the average differences in fluorescence intensity. The output from the microarray analysis was merged with the Unigene or Genebank descriptor and stored as an Excel (Microsoft Corp., Redmond, WA, USA) data spreadsheet. The definition of a 2-fold change, or no change in the expression of individual genes was predicated on the ranking of the difference call (DC) from two comparisons (2′1), namely, no change (NC) of expression for individual genes was merged with the Unigene or GeneBank descriptor and stored as an Excel data spreadsheet. The reproducibility of paired experiments was evaluated on the basis of the coefficient of variation (CV; SD/mean) for fold change (FC). The CV of FC must be ≤1.0. Finally, genes with a FC >2.0 were considered to be significant. These cut-off values represented a conservative estimate of the numbers of genes whose expression levels differed between samples. Gene categorization was based on a literature review. All data is MIAME compliant and that the raw data has been deposited in a MIAME compliant database (ArrayExpress, GEO), as detailed on the MGED Society website http://www.mged.org/Workgroups/MIAME/miame.html.

### De-Differentiation of Terminally Differentiated ATSC-Derived Bone and Fat, and Their Pluripotency

For the de-differentiation of fully differentiated ATSC into bone and fat, culture was developed at 37°C in a humidified atmosphere containing 5% CO_2_ with α-MEM containing 10% FBS and antibiotics. For de-differentiation, the culture plates were incubated in the hypoxic chambers and flushed with a gas mixture containing 1% O_2_, 5% CO_2_, and 94% N_2_ at 5–8 psi for 2 or 6 hours in DHP-derivative (10 ng/ml) containing culture medium at 37°C. The controls included parallel cultures in which the cells were exposed to normoxic conditions (21% oxygen tension). Finally, we verified the pluripotency of the de-differentiated cells via differential expressions of stemness genes, bone and fat markers, and cell growth-associated genes and the staining level of bone and fat after de-differentiation.

### Inhibition of De-Differentiated ATSC Cell Growth by Rex1 siRNA

Two complementary hairpin siRNA template oligonucleotides harboring the 21 nt target sequences of the human Rex1 were employed for transient transfection with Lipofectamine™2000 (Invitrogen) using 50 nM siRNA. Furthermore, the quantity of siRNA was optimized in accordance with the manufacturer's instructions. Three separate Rex1 siRNAs (Silencer® predesigned siRNAs; Ambion) and scrambled siRNAs with the same nucleotide content were assessed. When compared with unrelated control siRNAs and scrambled siRNAs, the Rex1 siRNAs resulted in an 80–90% reduction in Rex1 mRNA levels, as determined via real-time PCR. The siRNA that provided the most efficient inhibition (90–95%) was utilized for the experiments. For the detection of the inhibition of de-differentiated ATSC growth, we transfected Rex1 siRNA into de-differentiated ATSC counted dye-exclusive viable cells for 6 days.

### Bisulfite Modification and Sequencing of Genomic DNA

Genomic DNA was purified via phenol/chloroform/isoamylalcohol extraction, followed by one chloroform extraction, after which the DNA was ethanol-precipitated. The DNA was dissolved in distilled water. Bisulfite conversion was conducted using the EZ DNA Methylation–Gold kit (Zymo Research, USA), as described by the manufacturer. Briefly, unmethylated cytosines in DNA were converted into uracil via the heat-denaturation of DNA and with a specially designed CT conversion reagent. DNA was then desulphonated and subsequently cleaned and eluted. The bisulfite-modified DNA was then immediately utilized for PCR or stored at or below −20°C. The converted DNA was amplified via polymerase chain reaction (PCR) or designed with MethPrimer (http://www.urogene.org/methprimer) (Table S1). The PCR reactions were conducted in a MyGenie 96 Gradient Thermal Block (Bioneer, Daejeon, South Korea) in accordance with the following protocol: 95°C for 15 min, 40 cycles of 95°C for 20 sec, 43–58°C for 40 sec, 72°C for 30 sec, followed by an extension at 72°C for 10 min, and soaking at 4°C. The PCR products were cloned into bacteria (DH5α) by a pGEM T-Easy Vector System I (Promega, Madison, WI, USA). DNA extracted from bacterial clones was analyzed via sequencing with the M13 reverse primer (5′-AGCGGATAACAATTTCACACAGGA-3′), using an ABI 3730XL capillary DNA sequencer (Applied Biosystems) and represented as rows of circles, with each circle symbolizing the methylation state of one CpG.

### Induction of Rat Spinal Cord Injury and Cell Transplantation

Adult female Wistar rats weighing 250 g (∼5 weeks of age) were housed in a controlled environment and provided with standard rodent chow and water. Animal care was carried out in compliance with Korean regulations regarding the protection of animals used for experimental and other scientific purposes. The animals were subjected to traumatic spinal cord injury via a modified version of the protocol described by Kang et al. [32]. In brief, the spinous processes of T9 and T10 were removed with rongeurs, and a laminectomy was performed using a dental drill and rongeurs to expose the dorsal spinal cord in anesthetized rats. The dura was incised with microscissors and the dorsal and ventral columns, including the dorsal and ventral corticospinal tract (CST), were cut by lowering microscissors attached to a sterotaxic arm to a depth of 3.5 mm below the dorsal surface of the spinal cord and cutting twice. Experimental trials were first conducted to ascertain that this surgical procedure caused a consistent total transaction of the CST on both sides. For cell transplantation, GFP-lentivirus-labeled cells (1×10^6^ cells) were suspended in HBSS, mixed in accordance with the manufacturer's instructions with Matrigel (Gibco-BRL) and directly injected into the lesion site 1 week after injury. The control (vehicle) group received the same volume of HBSS mixed with Matrigel only. The rats were then randomly assigned into three groups: 15 spinal cord injury (SCI) rats receiving cell treatment (ATSC control or de-ATSC each) and 5 SCI rats as controls. A total of 35 animals were utilized in these experiments.

### Evaluation of Trans-Differentiation Properties of De-Differentiated Cells *In Vitro* and *In Vivo* Sci Rat Model

For the induction of neural differentiation, we cultured neurospheres in a neurobasal medium (NB; Invitrogen, Gaithersburg, MD, USA), supplemented with B27 (Invitrogen), 20 ng/ml of bFGF, and 10 ng/ml of EGF (Sigma) for 4–7 days. The culture density of the spheroid bodies was maintained at 20–50 cells/cm^2^ to prevent self-aggregation. Then, neurospheres derived from the cells were layered and cultured further on PDL-laminin double-coated well plates. To determine the expression of the neural markers, differentiated ATSC were fixed in 4% paraformaldehyde (PFA) fixative solution for 30 minutes at room temperature. After extensive washing in PBS, the cells were blocked for 30 minutes at room temperature with 1% normal goat serum. The cells were then incubated with primary antibodies against anti-TuJ 1 (1∶500; Sigma) and anti-GFAP (1∶1500; DAKO, USA). After extensive washing, the cells were incubated with FITC or Texas-Red conjugated secondary antibodies (1∶250; Molecular Probes, USA). We then analyzed the cells via fluorescence microscopy (Leica Microsystems, PA, USA). For cell-engrafted spinal cord tissue analysis, we performed immunohistochemistry as described in Materials and Methods S1.

### Electrophysiological Recording

Electrophysiological evaluation (evoked action potential of engrafted De-ATSCs) was performed before, immediately after and 30 days after the sciatic axotomy and De-ATSCs transplantation. Under anesthesia, the rat's left sciatic nerve and fourth digital nerve were exposed. Bipolar hooked platinum recording and stimulating electrodes were used to induce and record electrical activity. The stimulating electrode was placed under the proximal sciatic nerve and the recording electrode was placed under the fourth digital nerve. The evoked action potential in responding to the stimuli (one ms, 500 mV) in the ipsilateral sciatic nerve was recorded using Powerlab-800 system (AD Instruments, Colorado Springs, CO, http://www.adinstrumentsinc.com).

### Experimental Diabetes Induction and Cell Transplantation

Female C57BL6 mice, 8–10 weeks old were injected intraperitoneally (i.p.) with 50 mg/kg of body weight STZ (Sigma-Aldrich, St. Louis, Sigma) daily on five consecutive days. STZ was solubilized in sodium citrate buffer, pH 4.5, and injected within 15 minutes of preparation. And, control ATSCs (2×10^5^) and de-ATSCs (2×10^5^) were transplanted by tail vein injection on day 15 following STZ induction of diabetes (n = 8). Blood glucose was measured twice weekly with a glucometer (Accu-Chek Active blood glucose meter; F. Hoffmann-La Roche, Basel, Switzerland). Those animals were considered diabetic whose blood glucose level exceeded 10 mM at days 14 and 15. All animal procedures were approved by the Animal Care and Use Committee of the 21 century Frontier Stem Cells Research Center (South Korea).

### Statistical Analysis

All data were expressed as the means ± S.E.M from five or more independent experiments. The statistical significance of differences between groups was calculated via Student's two tailed *t*-test.

## Supporting Information

Materials and Methods S1 Supplementary materials and methods.
(0.06 MB DOC)

Figure S1 Evaluation of longevity and proliferation activity of dedifferentiated ATSC cells in long-term extended culture. (A) Cell proliferation activity was monitored by BrdU immunostaining at the specific passage of cultured de-ATSCs. (B) Viable cell counting was conducted via visual cell counts in conjunction with trypan blue exclusion. Data presented are presented as mean ±SD; n>4. * p% 0.05, and ** p% 0.01, Student's t test.
(4.19 MB TIF)

Figure S2 Function of Hypoxia/DHP-d in cell proliferation activity except apoptotic cell death signals in De-ATSC cells and TERT activity. (A) Verification of cell growth attenuation and exclusion of apoptotic cell death following de-ATSCs extended passage through cell proliferation and apoptotic signature analysis. (B) Comparative telomerase activities in de-ATSC cells, hES cell, and brain cancer cell lines, U87MG and A172.
(5.04 MB TIF)

Figure S3 Effects of Hypoxia/DHP-d exposure time on cell proliferation and differentiation efficiency of De-ATSCs. (A) Effects of Hypoxia/DHP-d exposure time schedule on cell proliferation after cell reprogramming. (B) Chondrogenic and Adipogenic differentiation efficiency in De-ATSCs compared to control ATSCs. Data presented are presented as mean ±SD; n>4. * p% 0.05, and ** p% 0.01, Student's t test.
(2.96 MB TIF)

Figure S4 Verification of genetic stability of dedifferentiated ATSCs through single nucleotide point (SNP) mutation analysis compared to control ATSCs.
(3.20 MB PDF)

Table S1 Bisulfite sequencing primers used in this study.
(0.07 MB DOC)
