# Supplementary material for: DHP-Derivative and Low Oxygen Tension Effectively Induces Human Adipose Stromal Cell Reprogramming
Source: PLoS One. 2010 Feb 9;5(2):e9026. doi: 10.1371/journal.pone.0009026 (PMC2817727; doi:10.1371/journal.pone.0009026)
Supplement: Materials and Methods S1 — Supplementary materials and methods. (0.06 MB DOC) [file pone.0009026.s001.doc]

**Supplementary materials and methods**

**Cell viability and proliferation assay.** Cell viability was assessed by visual cell counts in conjunction with trypan blue exclusion. Mitochondrial activity was assessed by measuring the ability of the cortical cultures to reduce 3,4,5-dimethyl thiazol-2-yl-2,5-diphenyl tetrazolium bromide (MTT; Sigma, St. Louis, MO, USA) to a colored formazan using a plate reader. In all viability assays, triplicate wells were established under each experimental condition, and each experiment was repeated at least three times. The raw data from each experiment were analyzed via analysis of variance with Fisher’s or t-tests. For flow cytometric analysis, cells were cultured in 100-mm dishes at densities that ensured exponential growth at the time of harvesting. Harvesting and processing protocols were used to detect DNA via flow cytometry with propidium iodide. The cells were analyzed with a BD Biosciences FACScan system (San Jose, CA, USA). The percentages of cells in the G0/G1, S, and G2/M phases of the cell cycle were determined using a DNA histogram fitting program (MODFIT; Verity Software, Topsham, ME, USA). A minimum of 10^4^ events/samples was collected. Clonogenic cell growth experiments were conducted with control ATSC and de-differentiated ATSC for the detection of colony forming units (CFU). Cells were grown in culture media for 10-15 days. The cells were then fixed in methanol and stained with methylene blue. Colonies containing >50 cells were evaluated via light microscopy and scored as survivors. In each case, the number of colonies were counted according to the number of cells plated multiplied by the plating efficiency.

**Real time RT-PCR and primer sets.** One week after the initiation of culturing, the total cellular RNA was extracted with Trizol (Life Technologies, Frederick, MA, USA) from cultured ATSC. Total RNA was reverse-transcribed into first-strand cDNA using an oligo-dT primer, then amplified via PCR using the indicated gene-specific primers (20 pM). The PCR reactions were conducted using an ABI 7700 Prism Sequence Detection System and SYBER green detection kit (Applied Biosystems, Foster, CA, USA). The primer sequences were designed with Primer Express software (PE-Applied Biosystems, Warrington, UK) using gene sequences obtained from the GeneBank database. The gene-specific primers were as follows: GAPDH: 5-CAT GAC CAC AGT CCA TGC CAT CAC T-3 and 5-TGA GGT CCA CCA CCC TGT TGC TGT A-3; Rex1: 5- TGA AAG CCC ACA TCC TAA CG-3 and 5- CAA GCT ATC CTC CTG CTT TGG-3; Oct-4: 5- CGC CAA CTG GCA TTG TCA T-3 and 5- TTC TCC TTG ATG TCA CGC AC-3; EGFR: 5- CTT CTT GCA GCG ATA CAG CTC-3 and 5- ATG CTC CAA TAA ATT CAC TGC-3; Nanog: 5- GCT GAG ATG CCT CAC ACG GAG-3 and 5- TCT GTT TCT TGA CTG GGA CCT TGT C-3; CDK2: 5- TCT CCC CTC CTC TTC CTT GT-3 and 5- CAG TGA AGC ACA ATG CCT GT-3; CDK1: 5- GGC TCT TGG AAA TTG AGC GGA-3 and 5- AGG AAC CCC TTC CTC TTC ACT-3, CDK4; 5- TGG TGT CGG TGC CTA TGG GA-3 and 5- GTC TAC CGT GAA TGT CGG CA-3; Cyclin-D2: 5- GAG AAG CTG TCC CTG ATC CGC AAG C-3 and 5- AGA CTT GGA GCC GTT GTG CTG CTC-3; Runx3: 5- CCG AGC CAT CAA GGT GAC CGT GGA C-3 and 5- GGG CTG GCT GCT GAA GTG GCT TGT-3; MMP2: 5- ACG ACC CGC ACA AGA ACT AT-3 and 5- CTG CAA AGA ACA CAG CCT TCT C; VEGF: 5- ACA TCT TCC AGG AGT ACC CTG ATG AG-3 and 5- GCA TTC ACA TTT GTT GTG CTG T-5; TERT: 5- AGC TAT GCC CGG ACC TCC AT-3 and 5- GCC TGC AGC AGG AGG ATC TT-3; and SOX2: 5**-**TAC CTC TTC CTC CCA CTC CA-3 and 5-ACT CTC CTC TTT TGC ACC CC-3.

**Flow cytometric analysis of surface epitopes.** For phenotypic characterization by flow cytometry, de-differentiated ATSC and cultured ATSC adherent cells were harvested via trypsinization, washed twice in PBS, and suspended at a concentration of 1X10^6^ cells/ml, then incubated with antibodies against the following antigens: CD117, CD34, CD90, CD164, CD133, and CD45 for 20 minutes. For FACS analysis, we utilized primary antibody conjugated directly with APC or FITC. Monoclonal antibodies against CD34 and CD45 were used to identify the cells as hematopoietic. The stained cells were then thoroughly washed in two volumes of PBS and fixed in neutralized 2% paraformaldehyde solution. For an isotype control, non-specific mouse or rabbit IgG (Dako, Chemicon, or Santa Cruz Biotechnology) was substituted for the primary antibody. The labeled cells were analyzed with a FACScan argon laser cytometer (Becton Dickinson, San Jose, CA, USA).

**Mesodermal and endodermal differentiation potencies of de-differentiated ATSC.** In order to compare the multipotential differentiation abilities of ATSC controls and de-differentiated ATSC, the cells were subjected to differentiation under known conditions to induce adipogenic, osteogenic, and chondrogenic lineages in human cells. Prior to culturing in the induction media, the cultures were grown to confluence. For adipogenic differentiation, the ATSC were induced by the passaging of cells at a dilution of 1:10 in control media supplemented with 10 ng/ml of insulin and 10^–9^ M dexamethasone (Sigma). Adipogenic differentiation was visualized by the presence of highly refractory intracellular lipid droplets via phase-contrast microscopy. In order to induce osteogenic differentiation, the cultures were fed daily for 3 weeks with control medium to which 10 mM β-glycerophosphate, 50 ng/ml ascorbic acid, and 10^–9^ M dexamethasone had been added. The mineralization of the extracellular matrix was visualized by the staining of the cultures with von Kossa and Alizarin Red. Von Kossa staining was conducted using an aqueous 5% AgNO_3_ solution, followed by 2 minutes of fixation in 5% Na_2_S_2_O_3_ solution. For chondrocyte differentiation, a pellet culture system was utilized. Approximately 3 x 10^6^ de-ATSC and ATSC controls were placed in wells of a 96-well plate. The pellet was cultured at 37ºC with 5% CO2 in 500 ul of chonodrogenic media containing 6.25 g/ml insulin, 10 ng/ml of transforming growth factor 1, and 50 ng of ascorbate-2-phosphate in control media for 2–3 weeks. The medium was replaced every 2 days for 15 days. For calcium deposit and chondrocyte analysis in paraffin-embedded tissue, we stained the specimens via the Masson and van Gieson staining methods. In order to evaluate *in vivo* differentiation potency, ATSC controls, and de-differentiated ATSC were immobilized in Matrigel (BD Bioscience). Approximately 2 x 10^6^ cells were mixed with Matrigel and subcutaneously implanted in 8-week-old immunodeficient beige mice (NIH III/bg/nu/xid; Charles River Laboratories, Wilmington, MA, USA). The procedures were conducted in accordance with the specifications of the approved protocol. The transplants were recovered 6 weeks later, fixed with 4% formalin, and decalcified with 10% EDTA (pH 8.0) for paraffin embedding. The paraffin-embedded sections were then deparaffinized and stained via Alzarin Red (bone), Masson (muscle and chondrocytes), and van Gieson (chondrocytes) staining. For beta-like cell differentiation, cells were cultured in “N2 media+NA” containing DMEM/F12 (Gibco-Invitrogen) supplemented with 10 mM nicotinamide (Sigma-Aldrich), ITS (1:50), B27 media supplement (1:50; Invitrogen), and 15% FBS. After 24 hours of culture, the medium was exchanged with high glucose (dextrose [3500 mg/L]) differentiation media for 2 weeks. After the induction of differentiation, we conducted immnocytochemistry using and insulin antibody.

**Preparation of tissue whole extracts and Western blot.** For the confirmation of differentially expressed proteins following the de-ATSC, the cultured cells were pooled and lysed in 500 *μ*l of lysis buffer (20 mM Tris-HCl [pH 7.5], 150 mM NaCl, 1 mM EDTA, 1% Triton X-100, 2.5 mM sodium pyrophosphate, 1 mM EGTA, 1 mM glycerophosphate, 1mM Na_3_VO_4_, and 1 mM PMSF). The lysates were clarified via 10 minutes of centrifugation at 15,000 x g and the total protein content was determined using a Bio-Rad protein assay kit (Millan, Italy). For Western blotting, equal amounts (40 *μ*g) of protein extracts in a lysis buffer were subjected to 10% SDS-PAGE analysis and transferred to nitrocellulose membranes. Anti-Nestin (1:500; Sigma), anti-MAP2ab (1:500; Sigma), anti-Tuj (1:500; Sigma), anti-HIF1α (1:500; Santa Cruz), anti-C-myc (1:300; Chemicon), anti-p53 (1:1000; BD Science), anti-p21 (1:1000; Santa Cruz), anti-acetyl H3 (1:2000; Upstate, USA), anti-acetyl H4 (1:10000; Upstate), anti-GFAP (1:3000; Dako), pSAPK/JNK (1:1000; Cell Signaling), anti-PLCgamma (1:500; Cell Signaling), anti-p-Raf (1:1000; Cell Signaling), anti-pERK (1:1000; Cell Signaling), anti-Jak2 (1:1000; Cell Signaling), anti-pSTAT 3 (1:1000; Cell Signaling), anti-Akt (1:1000; Cell Signaling), anti-GSKβ1 (1:1000; Cell Signaling), anti-MEKK (1:1000; Cell Signaling), anti-MEK (1:1000; Cell Signaling), anti-GAPDH (1:250; Chemicon), and anti-β-actin (1: 500; Sigma) antibodies were incubated with the membranes. The relative band intensities were determined using Quality-one 1-D Analysis software (Bio-Rad).

**Histologic analysis.** For immunohistochemical analysis of the frozen tissues, the sections were fixed for 30 minutes in 4% paraformaldehyde. The sections were then washed three times in PBS and nonspecific binding was blocked with 10% normal horse serum. The sections were then incubated overnight at 4°C with the following antibodies; anti-GFAP (1:2000; Dako), anti-Tuj (1:250; Sigma), and anti-NF160 (1:250; Sigma). After rinsing with primary antibodies, the sections were incubated for 1 hour. After extensive washing in PBS, the cells were incubated for 30 minutes with FITC or Texas–Red conjugated secondary antibodies (1:250; Molecular Probe; and 1:250; Jackson Laboratory, respectively). Controls in which the primary antibodies were omitted or replaced with irrelevant IgG resulted in no detectable staining. The specimens were evaluated using a Leica fluorescence microscope (Leica Microsystems, Exon, PA, USA). Immunocytochemical studies were repeated at least three times. The co-localization of CM-Dil with several neural lineage markers was performed via confocal microscopy using a Leica TCS sp2 laser scanning microscope (Leica Microsystems) equipped with 3 lasers. Double-labeled cells were verified via the collection of 1 μm sections through the slices. For the relative quantification of NF160- or TuJ-positive cells, area counts of the striatum were conducted. In each section, five adjacent fields were sampled, beginning where the upper and lower blades were joined. The average number of NF160- or TuJ-positive cells in five adjacent fields per section (control animals, n=3; ATSC control or de-ATSC animals, n=5 each) was plotted, along with the percentage of NF160- or TuJ-positive cells per section. Error bars represent standard deviations.

**Cell migration assay.** In order to assess the migration activity of the dedifferentiated ATSC *in vitro*, the cells were transferred to culture dishes containing low serum (1%) growth medium. The cultured cells were transferred into transwell membranes (Costar, 8 μm pore size), coated on both sides with laminin, and placed in 6-well plates. In the upper chamber, 200-300 μl of dedifferentiated ATSC or control ATSC were preincubated for 2 hours and cultured overnight at 37°C in a CO_2_ incubator. For analysis, migrating cells on the lower surface were air-dried and counterstained for 20 minutes with Harris hematoxylin and washed. The stained inserts were positioned on object slides and the numbers of cells on the lower surfaces were assessed at x200 in an inverted bright-field microscope. Ten x20 fields per insert were counted. In this study, migration is expressed as a percentage of the cells per field of spontaneous migration toward the cell bottom.

**Supplementary Figure Legend**

**Figure S1. Evaluation of longevity and proliferation activity of dedifferentiated ATSC cells in long-term extended culture.** (A) Cell proliferation activity was monitored by BrdU immunostaining at the specific passage of cultured de-ATSCs. (B) Viable cell counting was conducted via visual cell counts in conjunction with trypan blue exclusion. Datas presented are presented as mean ±SD; *n>4*. * p % 0.05, and ** p % 0.01, Student’s t test.

**Figure S2. Function of Hypoxia/DHP-d in cell proliferation activity except apoptotic cell death signals in De-ATSC cells and TERT activity.** (A) Verification of cell growth attenuation and exclusion of apoptotic cell death as following de-ATSCs extended passaging through cell proliferation and apoptotic signature analysis. (B) Comparative telomerase activities in de-ATSC cells, hES cell, and brain cancer cell lines, U87MG and A172.

**Figure S3. Effects of Hypoxia/DHP-d exposure time on cell proliferation and differentiation efficiency of De-ATSCs**. (A) Effects of Hypoxia/DHP-d exposure time schedule on cell proliferation after cell reprogramming. (B) Chondrogenic and Adipogenic differentiation efficiency in De-ATSCs compare to control ATSCs. Datas presented are presented as mean ±SD; *n>4*. * p % 0.05, and ** p % 0.01, Student’s t test.

**Figure S4. Verification of genetic stability of dedifferentiated ATSCs through single nucleotide point (SNP) mutation analysis compare to control ATSCs.**
