# Supplementary figures and images for: DHP-Derivative and Low Oxygen Tension Effectively Induces Human Adipose Stromal Cell Reprogramming
Source: PLoS One. 2010 Feb 9;5(2):e9026. doi: 10.1371/journal.pone.0009026 (PMC2817727; doi:10.1371/journal.pone.0009026)

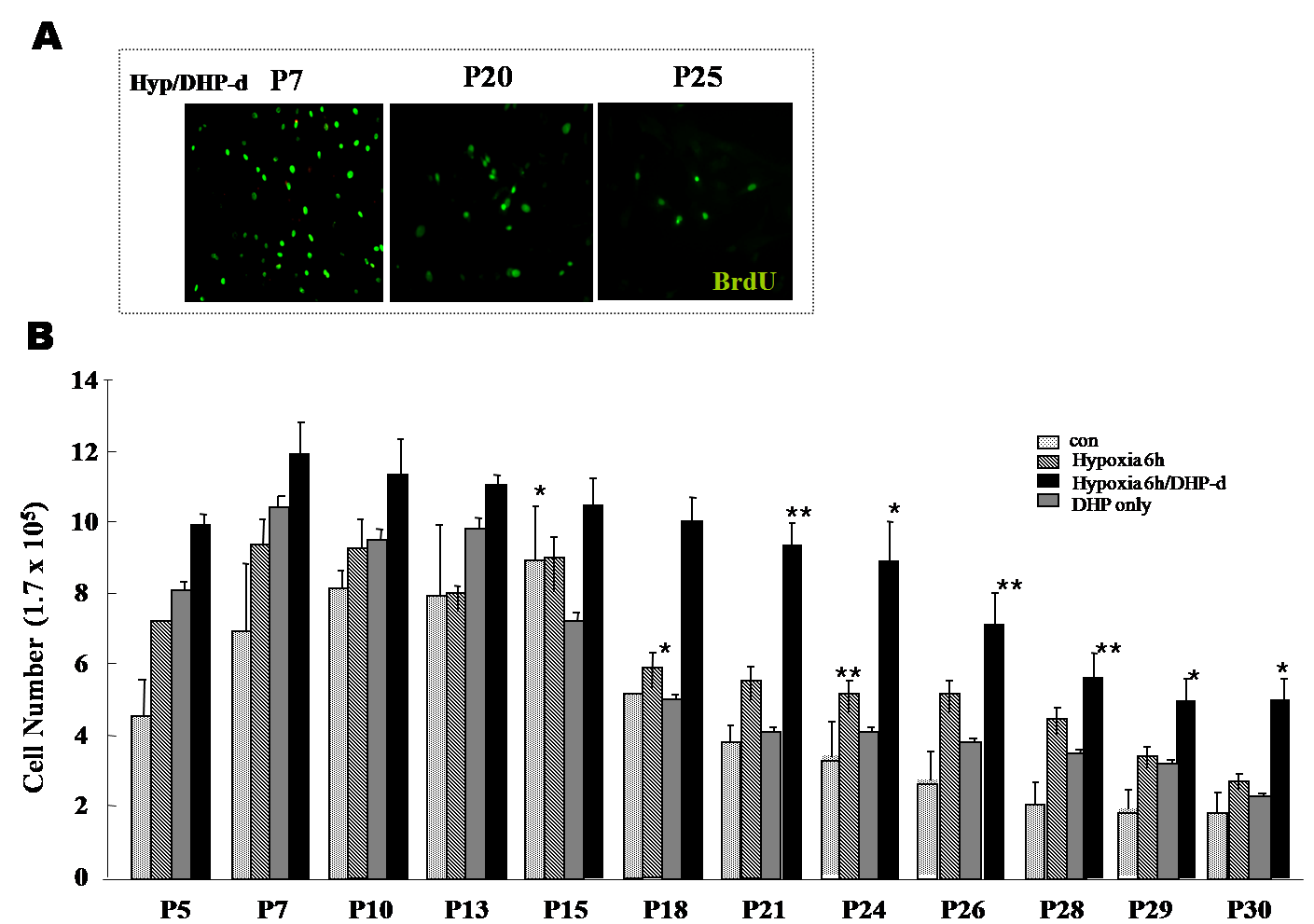

Supplement: Figure S1 — Evaluation of longevity and proliferation activity of dedifferentiated ATSC cells in long-term extended culture. (A) Cell proliferation activity was monitored by BrdU immunostaining at the specific passage of cultured de-ATSCs. (B) Viable cell counting was conducted via visual cell counts in conjunction with trypan blue exclusion. Data presented are presented as mean ±SD; n>4. * p% 0.05, and ** p% 0.01, Student's t test. (4.19 MB TIF) [file pone.0009026.s002.tif]

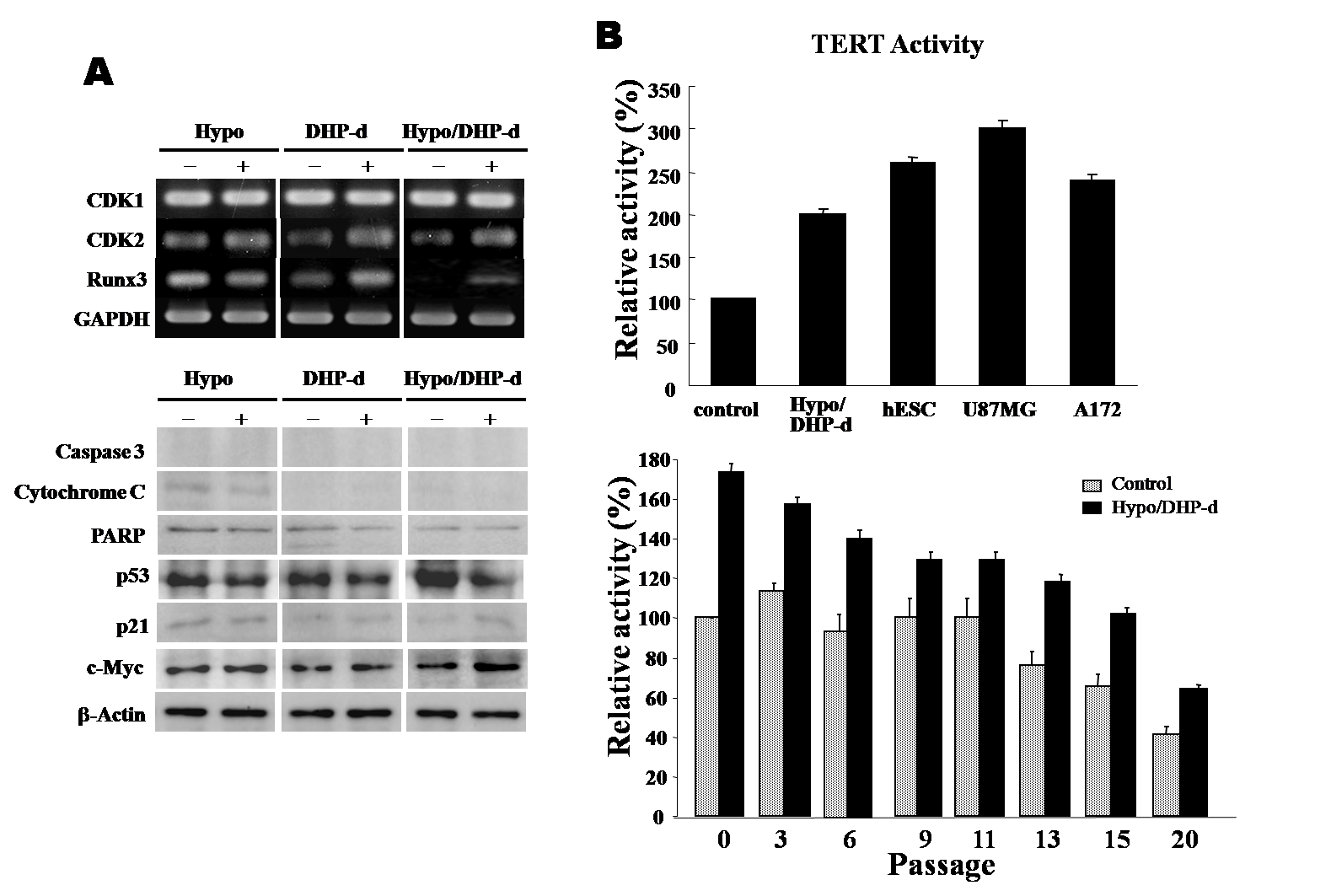

Supplement: Figure S2 — Function of Hypoxia/DHP-d in cell proliferation activity except apoptotic cell death signals in De-ATSC cells and TERT activity. (A) Verification of cell growth attenuation and exclusion of apoptotic cell death following de-ATSCs extended passage through cell proliferation and apoptotic signature analysis. (B) Comparative telomerase activities in de-ATSC cells, hES cell, and brain cancer cell lines, U87MG and A172. (5.04 MB TIF) [file pone.0009026.s003.tif]

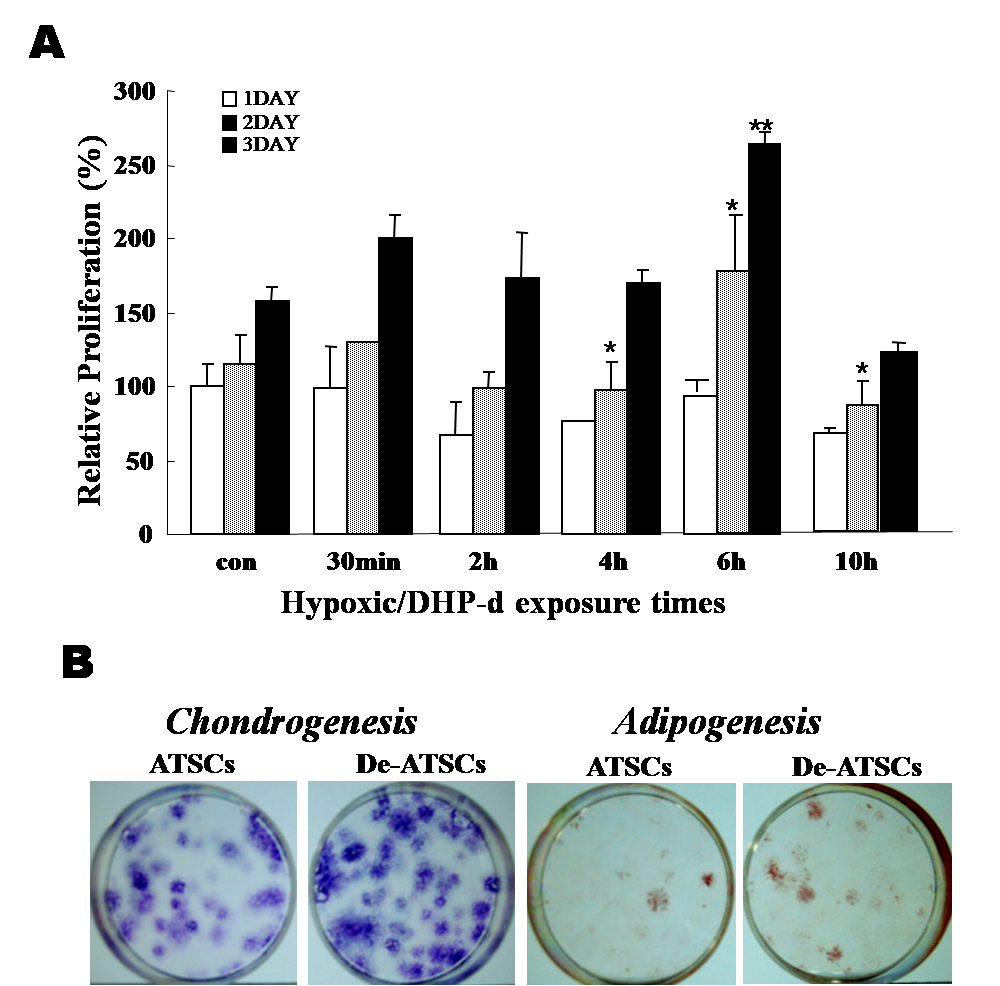

Supplement: Figure S3 — Effects of Hypoxia/DHP-d exposure time on cell proliferation and differentiation efficiency of De-ATSCs. (A) Effects of Hypoxia/DHP-d exposure time schedule on cell proliferation after cell reprogramming. (B) Chondrogenic and Adipogenic differentiation efficiency in De-ATSCs compared to control ATSCs. Data presented are presented as mean ±SD; n>4. * p% 0.05, and ** p% 0.01, Student's t test. (2.96 MB TIF) [file pone.0009026.s004.tif]
