# Supplementary material for: DHP-Derivative and Low Oxygen Tension Effectively Induces Human Adipose Stromal Cell Reprogramming
Source: PLoS One. 2010 Feb 9;5(2):e9026. doi: 10.1371/journal.pone.0009026 (PMC2817727; doi:10.1371/journal.pone.0009026)

SFig. 4

Chromosome 1~4

hATSC_con **De-ATSC_**

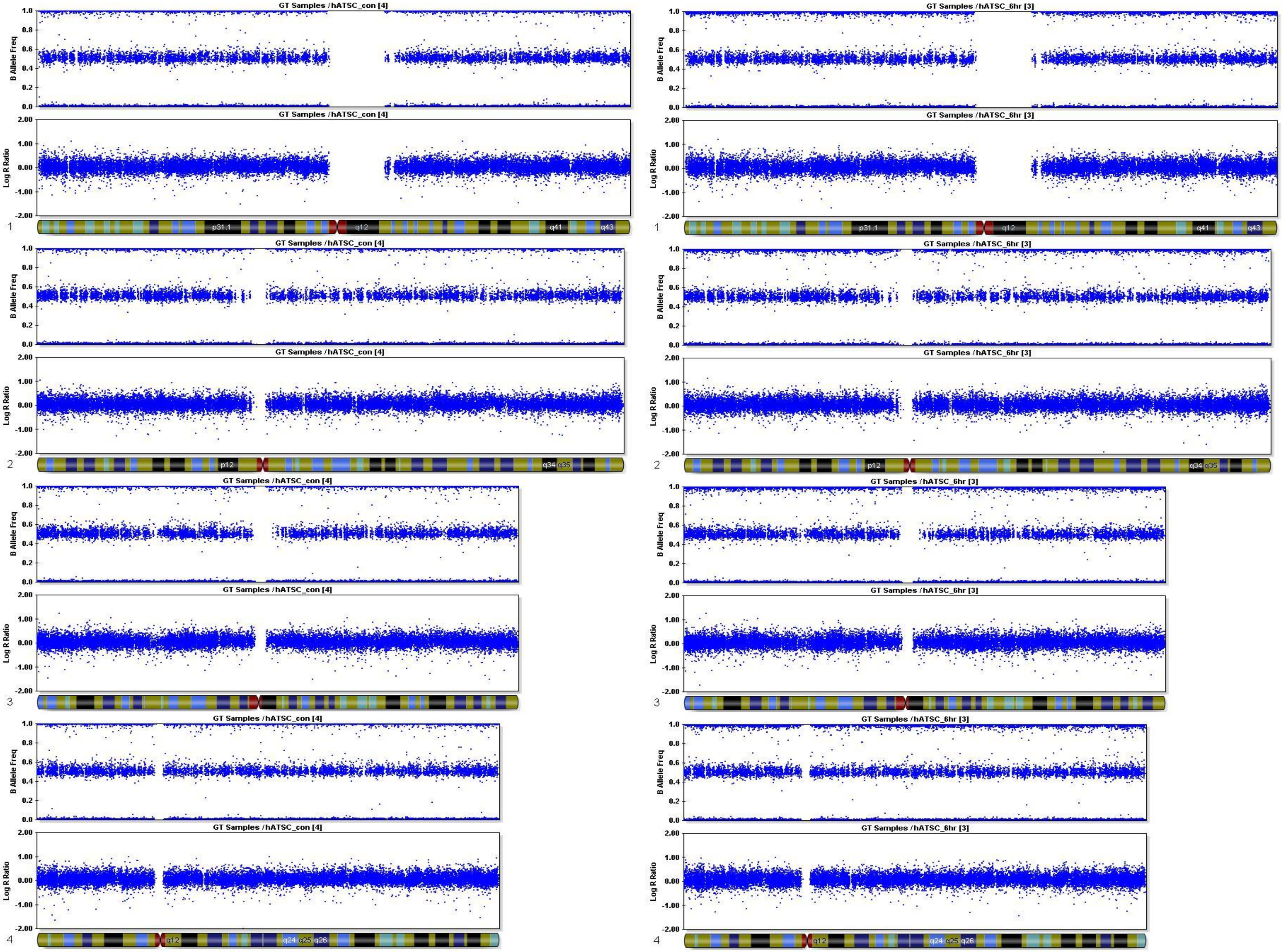

Chromosome 5~8

hATSC_con

De-hATSC_

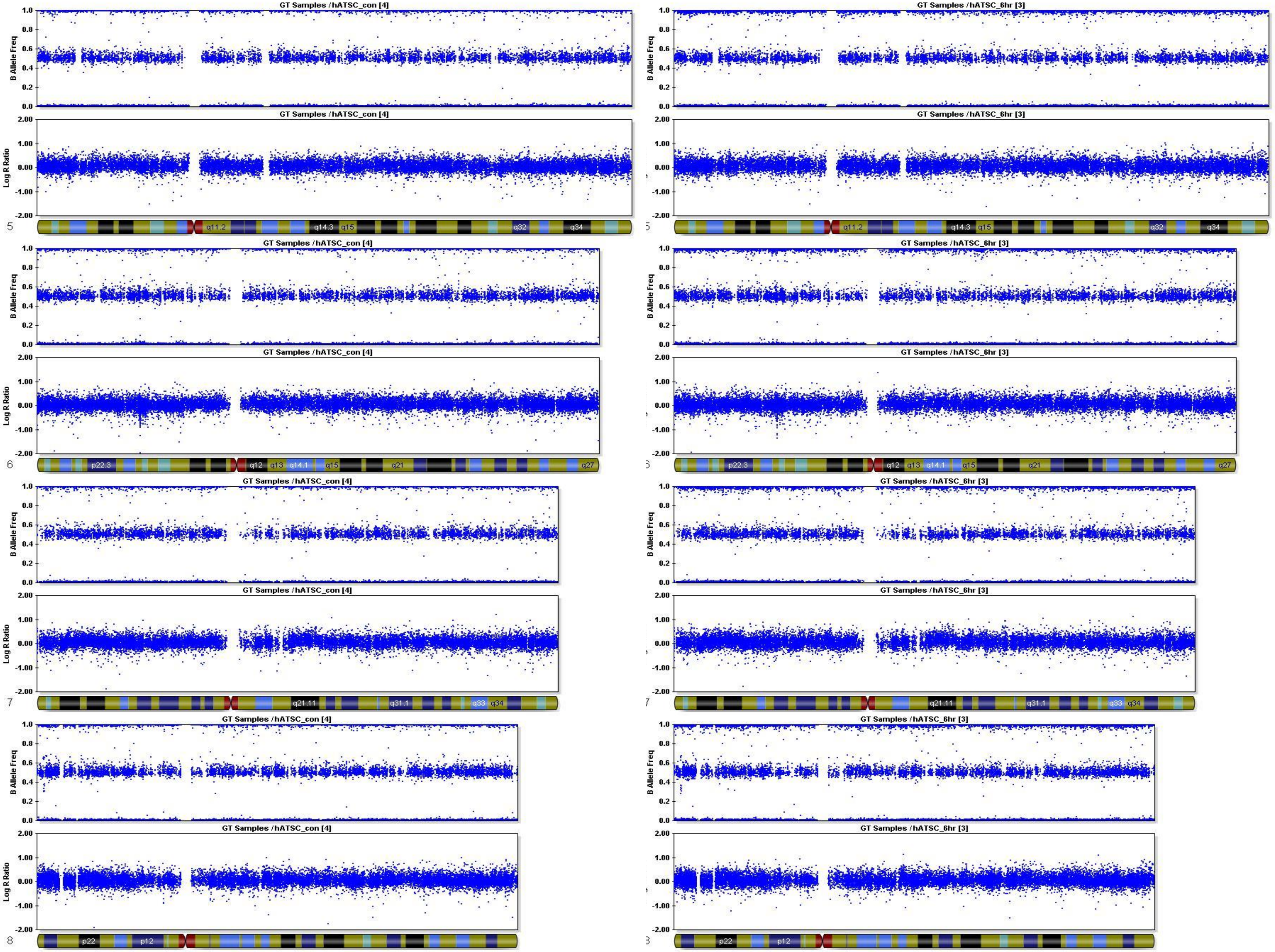

Chromosome 9~12

hATSC_con

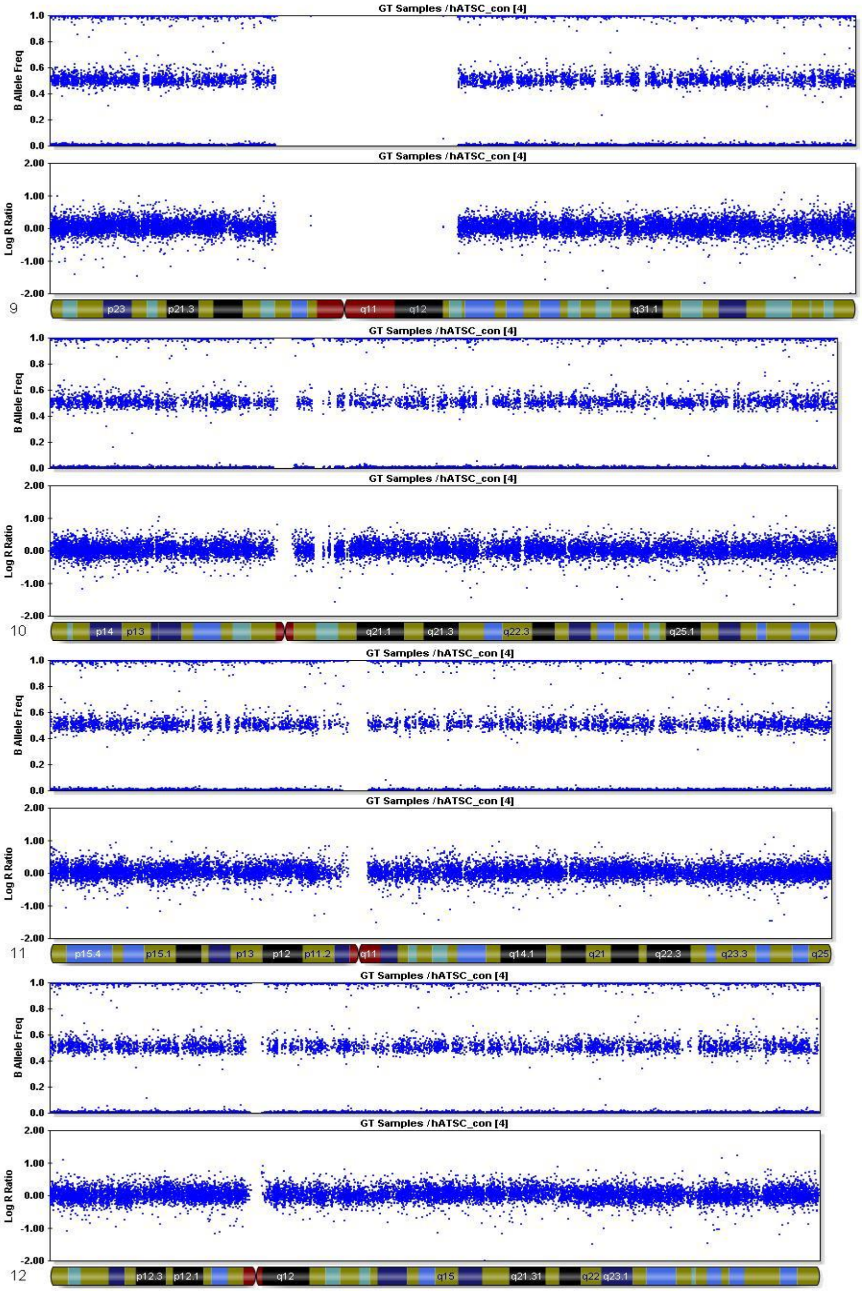

De-hATSC

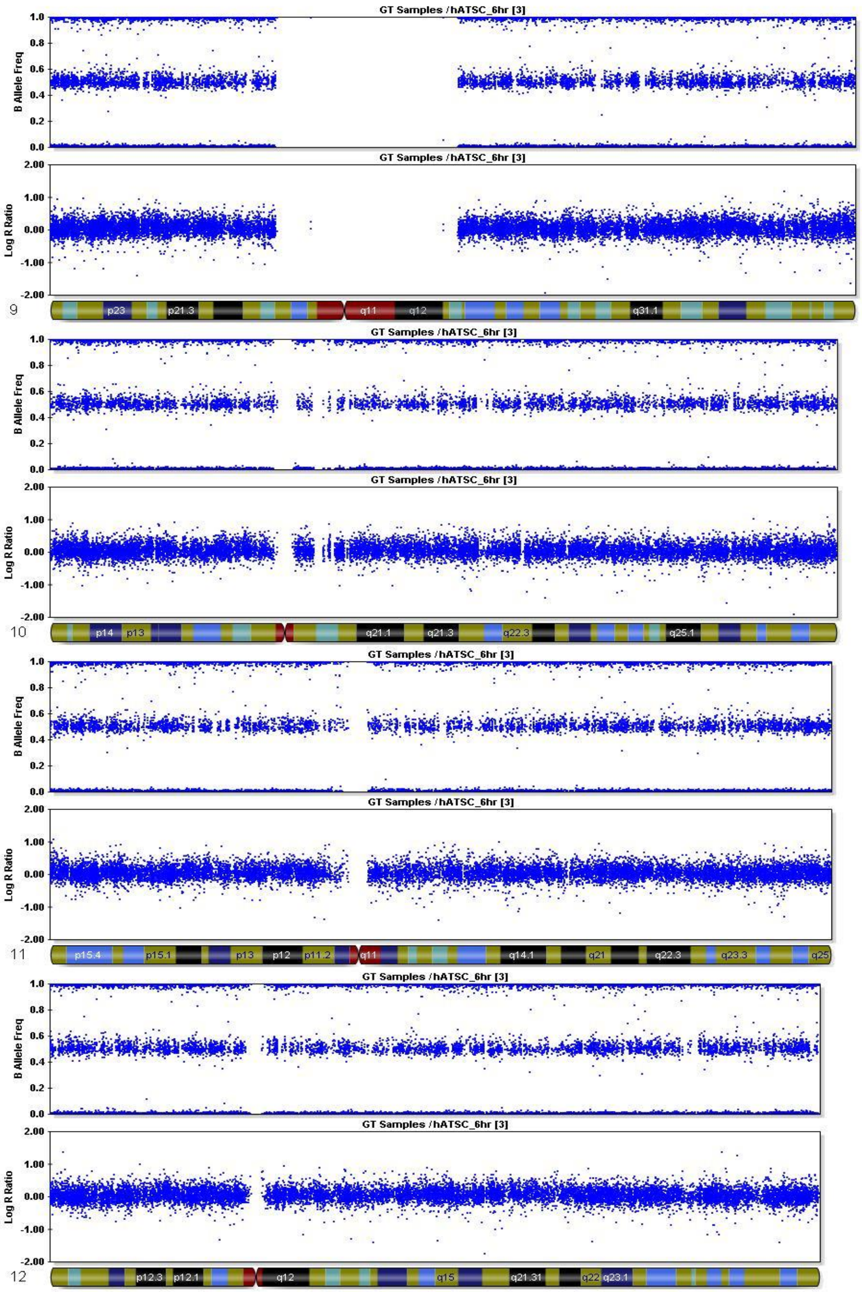

Chromosome 13~16

hATSC_con

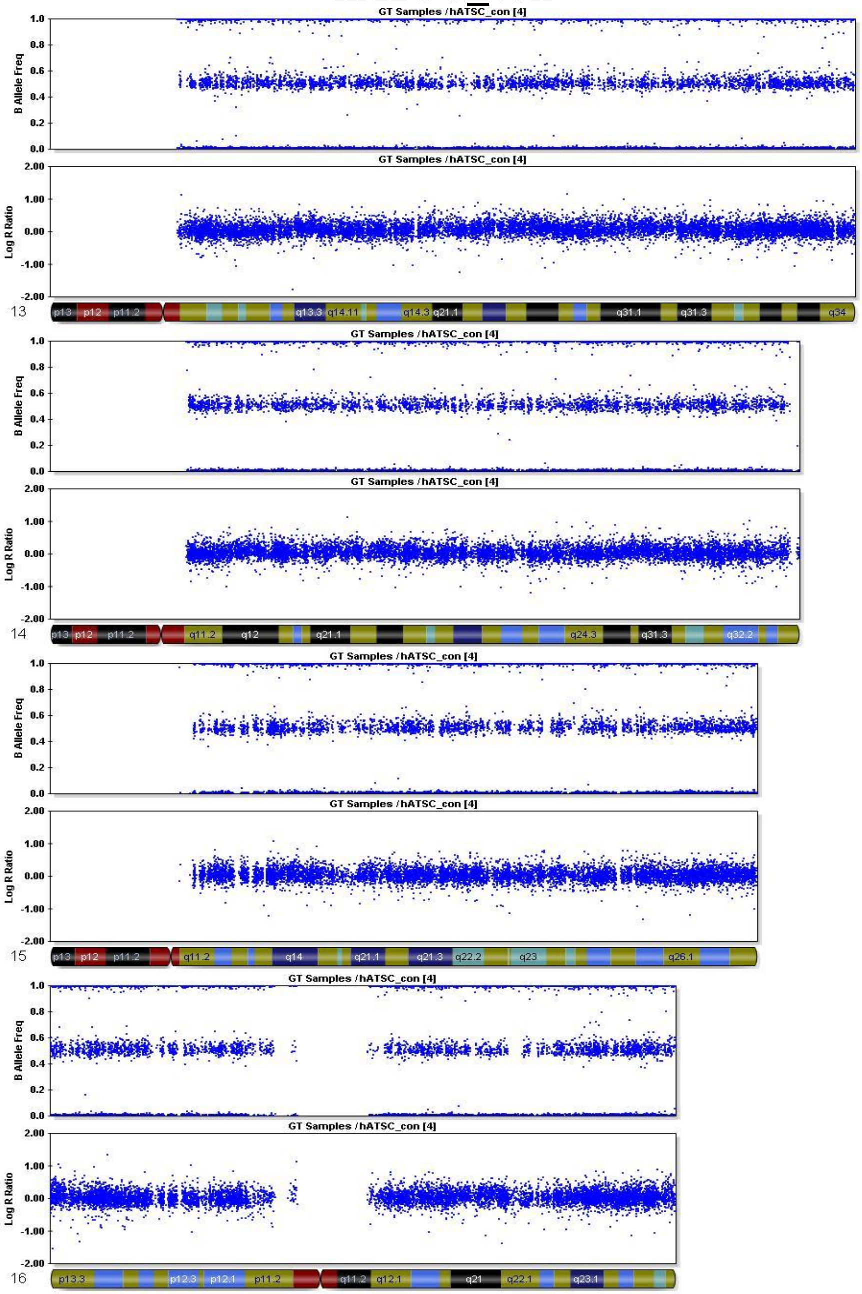

De-hATSC_

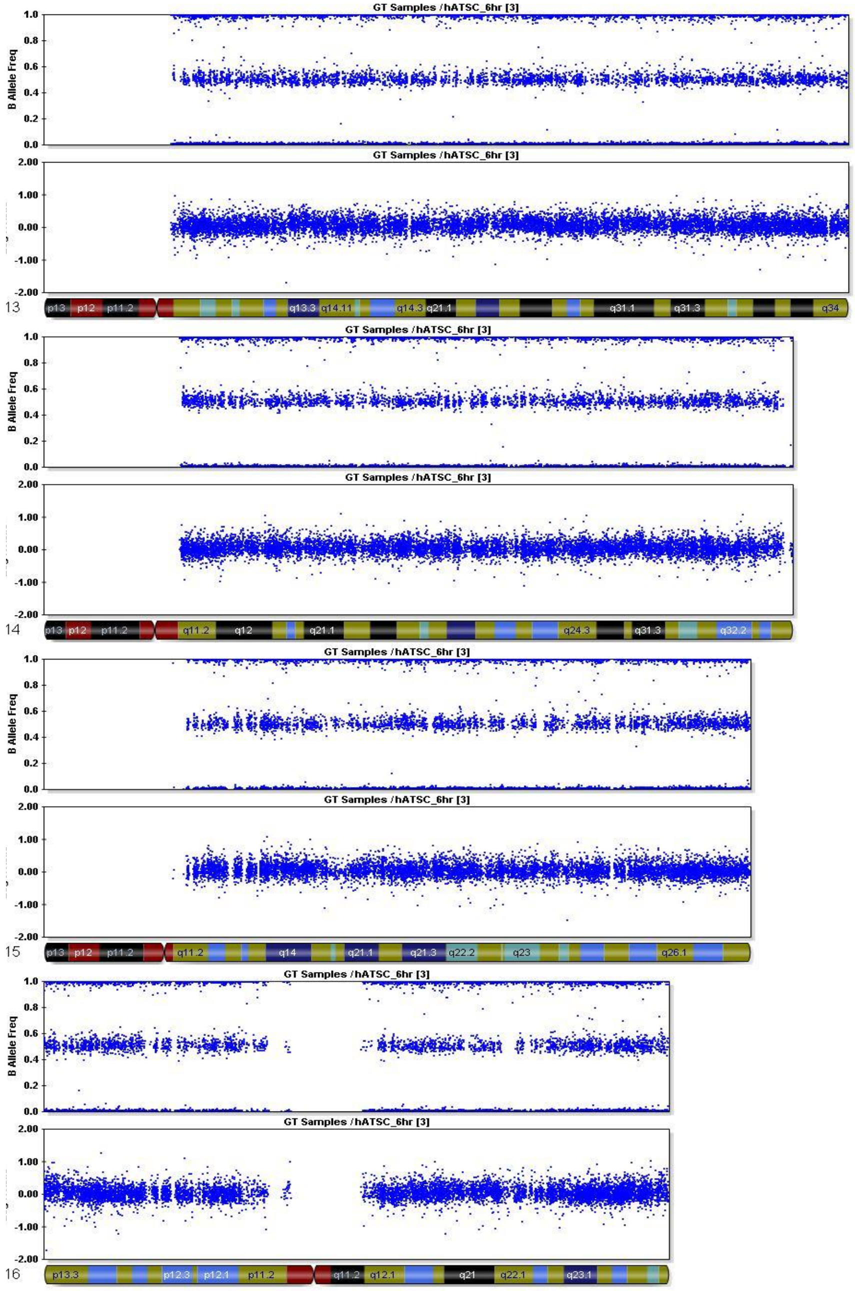

Chromosome 17~20

hATSC_con

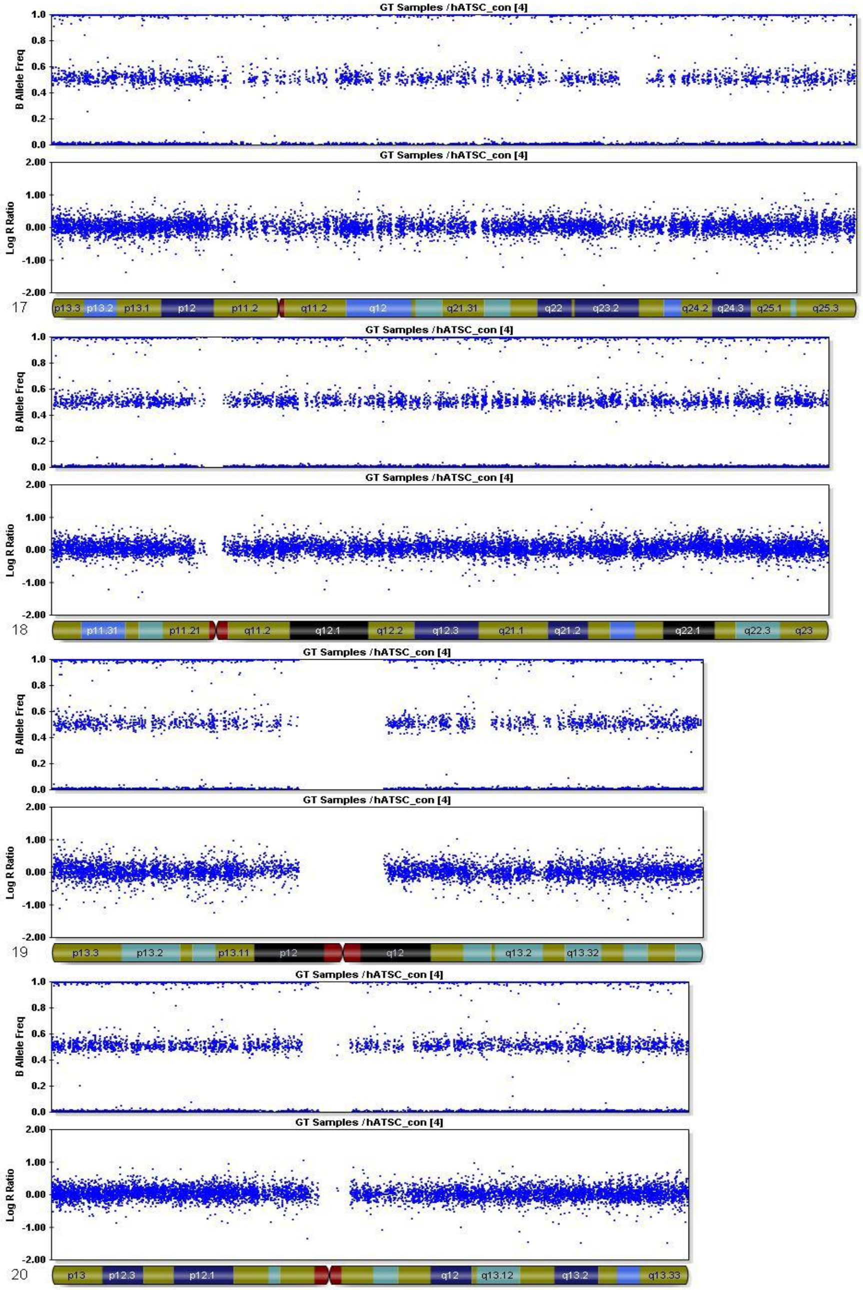

De-hATSC_

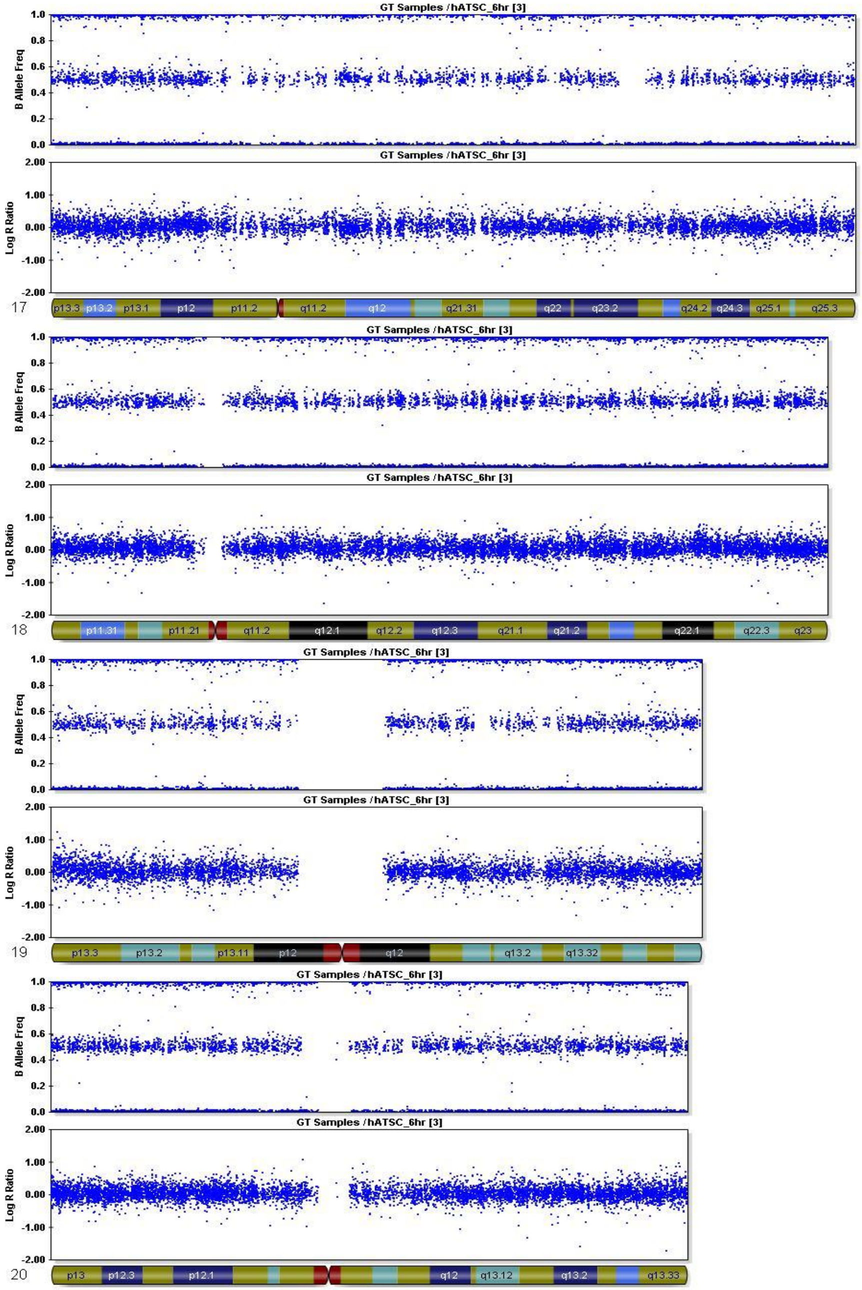

Chromosome 21~X

hATSC_con

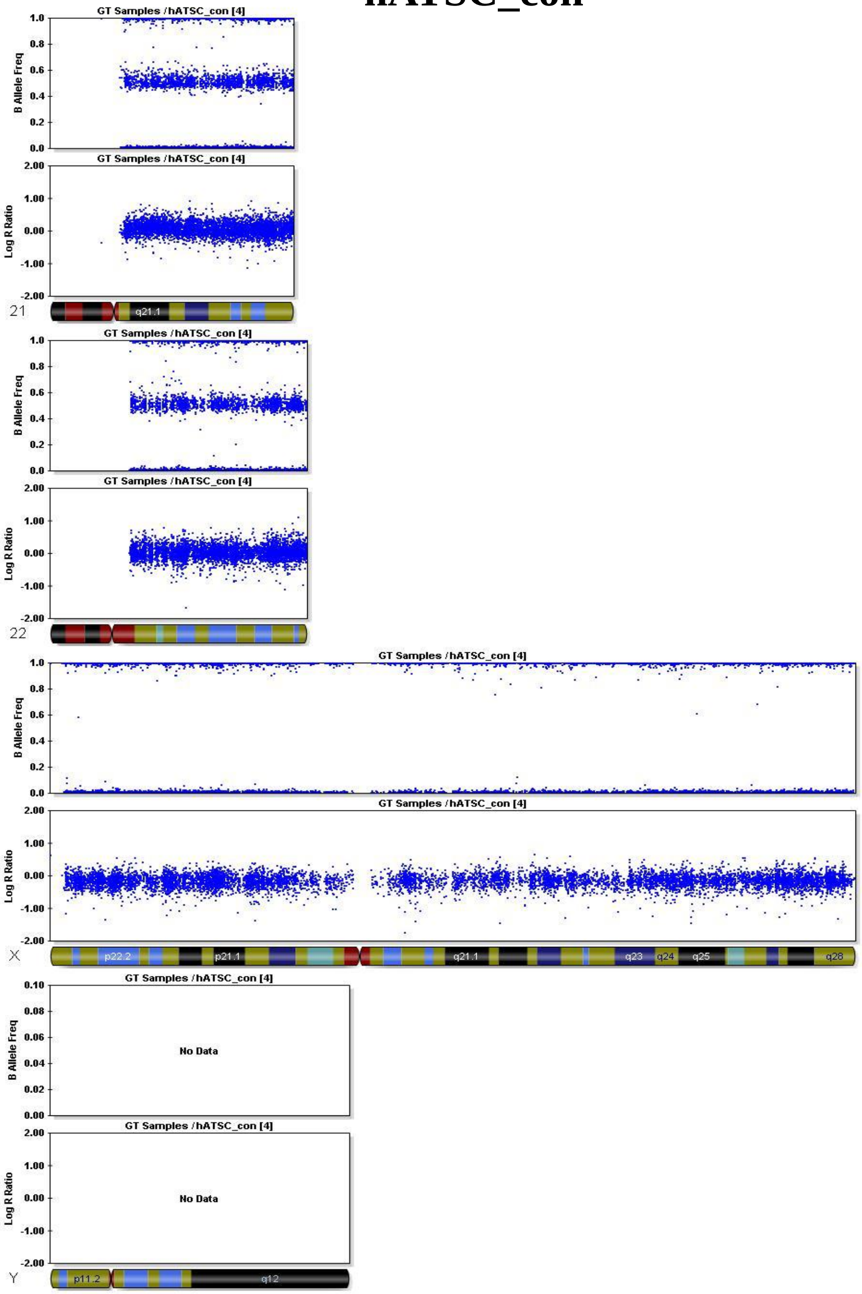

De-hATSC

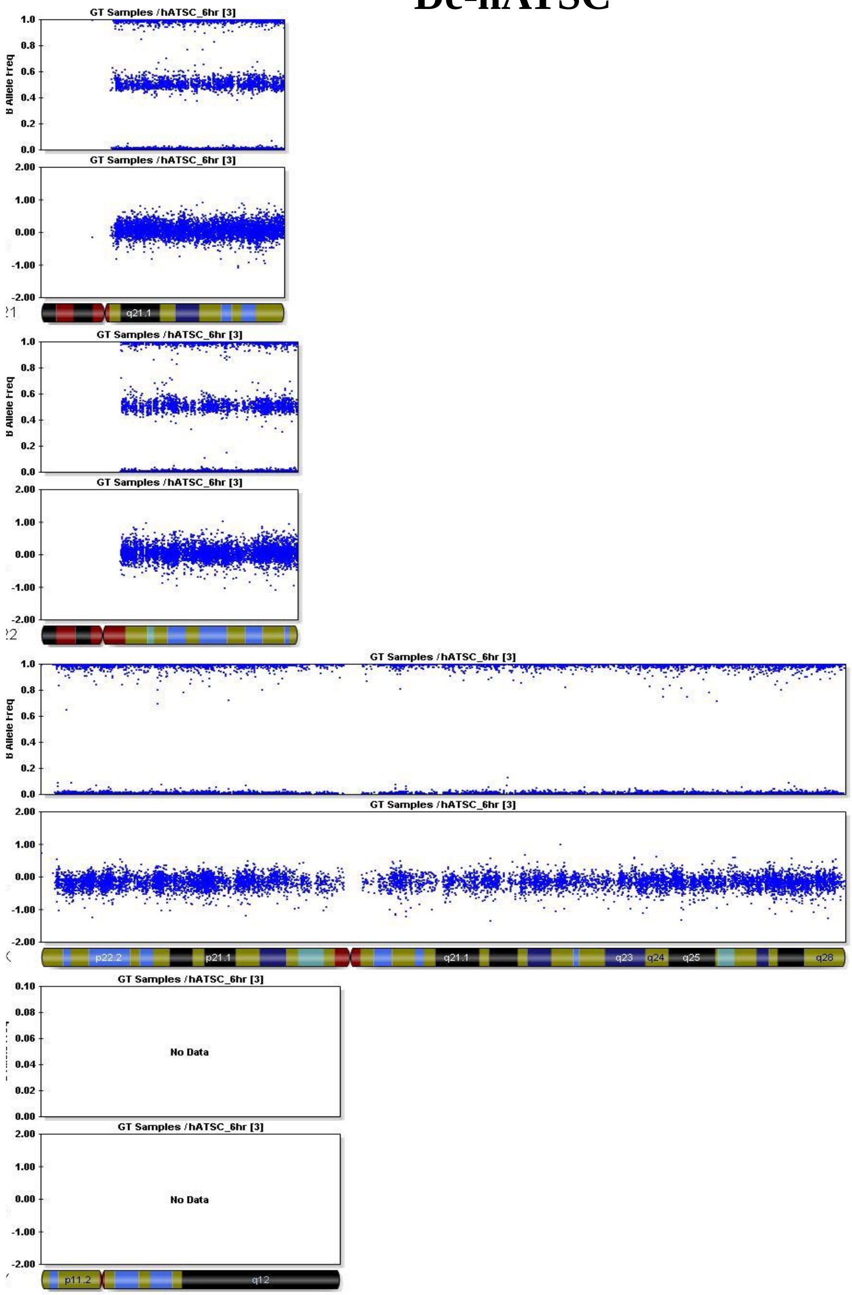

Supplement: Figure S4 — Verification of genetic stability of dedifferentiated ATSCs through single nucleotide point (SNP) mutation analysis compared to control ATSCs. (3.20 MB PDF) [file pone.0009026.s005.pdf]
