# Supplementary material for: DHP-Derivative and Low Oxygen Tension Effectively Induces Human Adipose Stromal Cell Reprogramming
Source: PLoS One. 2010 Feb 9;5(2):e9026. doi: 10.1371/journal.pone.0009026 (PMC2817727; doi:10.1371/journal.pone.0009026)
Supplement: Table S1 — Bisulfite sequencing primers used in this study. (0.07 MB DOC) [file pone.0009026.s006.doc]

**Table S1** Bisulfite sequencing primers used in this study.

| Gene name | Primer pair | Forward primer [F] Reverse primer [R] | Seq. coverage relative to TSS | Annealing temp. (℃) |
| --- | --- | --- | --- | --- |
| OCT4 | OCT4_1 | F : TTTTTAGTTTTTTTTAGGTTTAA ^a^  R : TAAACAAAAAACCCATTCCC ^a^ | -2995 to -2723 | 51 |
|  | OCT4_2 | F : TTAGGAAAATGGGTAGTAGGGATTT ^a^  R : TACCCAAAAAACAAATAAATTATAAAACCT ^a^ | -2609 to -2417 | 58 |
|  | OCT4_3 | F : ATTTGTTTTTTGGGTAGTTAAAGGT ^a^  R : CCAACTATCTTCATCTTAATAACATCC ^a^ | -2344 to -2126 | 58 |
|  | OCT4_4 | F : GGATGTTATTAAGATGAAGATAGTTGG ^a^  R : CCTAAACTCCCCTTCAAAATCTATT ^a^ | -2136 to -1721 | 58 |
|  | OCT4_5 | F : GAAGGGGAAGTAGGGATTAATTTT ^a^ R : CAACAACCATAAACACAATAACCAA ^a^ | -1014 to -720 | 58 |
|  | OCT4_6 | F : TAGTTGGGATGTGTAGAGTTTGAGA ^a^ R : TAAACCAAAACAATCCTTCTACTCC ^a^ | -567 to -309 | 58 |
|  | OCT4_7 | F1 : ATAAAGTGAGATTTTGTTTTAAAAA ^b^  R1 : AACATAAAAAAATCCCCCACAC ^b^ F2 : GGGATTTGTATTGAGGTTTTGG ^b^  R2 : CCCACACCTCAAAACCTAAC ^b^ | -202 to +231 | 50  56 |
| NANOG | NANOG_1 | F : AGAGATAGGAGGGTAAGTTTTTTTT ^a^ R : ACTCCCACACAAACTAACTTTTATTC ^a^ | -1503 to -1254 | 58 |
|  | NANOG_2 | F : GAGTTAAAGAGTTTTGTTTTTAAAAATTAT ^a^  R : TCCCAAATCTAATAATTTATCATATCTTTC ^a^ | -1203 to -911 | 52 |
|  | NANOG_3 | F : TTAATTTATTGGGATTATAGGGGTG ^a^ R : AACAACAAAACCTAAAAACAAACC ^a^ | -334 to -163 | 58 |
| SOX2 | SOX2_1 | F : GTAGGTTGGTTTTGGGAGTTTTT R : AATTAATAAACAACCATCCATATAAC | -268 to -52 | 55 |
|  | SOX2_2 | F : TGTTTTTTTAAGATTAGGATTGAGAGAA R : AAAACAAACTAAAATCAAAATCAAA | +80 to +253 | 53 |
|  | SOX2_3 | F : ACAAACTAACTCTAAAAACC ^c^ R : GGTTGTTAGGGAATAAATGG ^c^ | +427 to +616 | 43 |
|  | SOX2_4 | F : AGATGGTTTAGGAGAATTTTAAGATGTATA R : AACCCAACTAATCCTACATCATACTATAAC | +600 to +965 | 55 |
|  | SOX2_5 | F : GGTAGTTATAGTATGATGTAGGATTAGTTG R : AACCCATAAAACCAAAAACCATA | +932 to +1151 | 55 |
|  | SOX2_6 | F : GGGATATGATTAGTATGTATTTTTT R : AATTTTCTCCATACTATTTCTTACTCTCCT | +1239 to +1482 | 57 |
| REX1 | REX1_1 | F : AAATATTGGGGGTGTTTGAAATAAT R : CCCAACTACTCAAAAAACTAAAACAA | -868 to -590 | 57 |
|  | REX1_2 | F : AAAAGGGTAAATGTGATTATATTTA R : CAAACTACAACCACCCATCAAC | -423 to -68 | 54 |
|  | REX1_3 | F : ATGGGTGGTTGTAGTTTGATTAGAT R : TTTCAACATTTAAAACCAATAACCAA | -85 to +279 | 57 |
|  | REX1_4 | F : TTATTATAAAAGAGTTAGGAAGTTTGTATA R : ATTACCCAAACTAAAATACAACAAC | +2229 to +2547 | 54 |
|  | REX1_5 | F : TTTGGAGGAATATTTGGTATTGATT R : CCTATTACAACCTTAAAAAAAACACAC | +7497 to +7889 | 51 |
| TERT | TERT | F : CTACCCCTTCACCTTCCAA ^d^ R : GTTAGTTTTGGGGTTTTAGG ^d^ | -151 to +164 | 57 |
| VEGFA | VEGFA_1 | F : GTTATTATAGGGAAGTTGGGTGAAT R : CCAAAATTCACAACCTAAAAATTAC | -569 to -212 | 57 |
|  | VEGFA_2 | F: TAGGTTGTGAATTTTGGTGGGG R : AAATAAAACAATCTCCCCAAACC | -228 to +272 | 57 |
|  | VEGFA_3 | F: AGGTAGTAAGAGTTTTAGAGAGAAGT R : AAAACAACCCAAAAATTAAAC | -317 to +677 | 53 |
|  | VEGFA_4 | F : TGGGTGTATTGGAGTTTTGTTTTGTT R : CCCTACCCACTAATCTCTAACTCCC | +1050 to +1502 | 57 |
| NESTIN | NESTIN_1 | F : ATTTAATATTTTTTGGTAGGGGGTG R : CCTAAAAAACAAAAACAAATATCTAATATT | -808 to -542 | 53 |
|  | NESTIN_2 | F : GTTTGTTTATTTTTAGTGGGTTAGA R : CTCCAACTCTTCAACCAAATTATC | +101 to +400 | 55 |
| RUNX3 | RUNX3_1 | F : ATTTTGGAGGATTTGTTTTGGG ^e^ R : CAACCTACCCRACTAATCCC ^e^ | -391 to -201 | 54 |
|  | RUNX3_2 | F : TTAYGAGGGGYGGTYGTAYGYG ^f^ R : AAAACRACCRACRCRAACRCCTCC ^f^ | -263 to -43 | 56 |
| CDK2 | CDK2_1 | F : TTAAAGTAGGTATTTGGGAAGAG R : TTTAACCAACTTAAAACAATATTACC | -176 to +75 | 58 |
|  | CDK2_2 | F : TTTAAGTTGGTTAAATTGATAAGAG R : ATTAAAAAAAACAATCAAAAAAATCC | +61 to +410 | 54 |
| TP53 | TP53 | F : AAAAATTGAAGTTTATAGAGGTTAAGGGT R : CACAAACCCAAAATAAAACCAATAC | +12901 to +13298 | 58 |

^a^ From Freberg *et al.* (2007).

^b^ From Shen *et al.* (2006).

^c^ From Yeo *et al.* (2007).

^d^ From Renaud *et al.* (2007).

^e^ From Kim *et al.* (2004).^f^

From Ku *et al.* (2004).
